# Discovering and Constructing ceRNA-miRNA-Target Gene Regulatory Networks during Anther Development in Maize

**DOI:** 10.3390/ijms20143480

**Published:** 2019-07-15

**Authors:** Ziwen Li, Xueli An, Taotao Zhu, Tingwei Yan, Suowei Wu, Youhui Tian, Jinping Li, Xiangyuan Wan

**Affiliations:** 1 Biology and Agriculture Research Center, University of Science and Technology Beijing, Beijing 100024, China; 2 Beijing Engineering Laboratory of Main Crop Bio-Tech Breeding, Beijing International Science and Technology Cooperation Base of Bio-Tech Breeding, Beijing Solidwill Sci-Tech Co. Ltd., Beijing 100192, China

**Keywords:** microRNA, competing endogenous RNA (ceRNA), ceRNA-miRNA-target gene regulatory network, anther development, *Zea mays*

## Abstract

The “competing endogenous RNA (ceRNA) hypothesis” has recently been proposed for a new type of gene regulatory model in many organisms. Anther development is a crucial biological process in plant reproduction, and its gene regulatory network (GRN) has been gradually revealed during the past two decades. However, it is still unknown whether ceRNAs contribute to anther development and sexual reproduction in plants. We performed RNA and small RNA sequencing of anther tissues sampled at three developmental stages in two maize lines. A total of 28,233 stably transcribed loci, 61 known and 51 potentially novel microRNAs (miRNAs) were identified from the transcriptomes. Predicted ceRNAs and target genes were found to conserve in sequences of recognition sites where their corresponding miRNAs bound. We then reconstructed 79 ceRNA-miRNA-target gene regulatory networks consisting of 51 known miRNAs, 28 potentially novel miRNAs, 619 ceRNA-miRNA pairs, and 869 miRNA-target gene pairs. More than half of the regulation pairs showed significant negative correlations at transcriptional levels. Several well-studied miRNA-target gene pairs associated with plant flower development were located in some networks, including *miR156*-*SPL*, *miR159*-*MYB*, *miR160*-*ARF*, *miR164*-*NAC*, *miR172*-*AP2*, and *miR319*-*TCP* pairs. Six target genes in the networks were found to be orthologs of functionally confirmed genes participating in anther development in plants. Our results provide an insight that the ceRNA-miRNA-target gene regulatory networks likely contribute to anther development in maize. Further functional studies on a number of ceRNAs, miRNAs, and target genes will facilitate our deep understanding on mechanisms of anther development and sexual plants reproduction.

## 1. Introduction

Anther development is a crucial biological process for male fertility and sexual reproduction in plants. During the past decades, many genic male sterility (GMS) genes in this process and their underlying molecular mechanisms have been identified and studied in the model plant *Arabidopsis* as well as important crops such as rice, maize, wheat, and soybean [1,2,3,4]. In general, anther development is a complex process that encompasses many key stages from floral identity to the production of mature pollen grains, during which GMS genes involved in signal transduction, cell division, apoptosis, metabolism, and transport are temporally and spatially activated or repressed by finely-tuned regulatory pathways [1,5,6,7]. All the GMS genes investigated previously, and their regulatory relationships, together comprise a gene regulatory network (GRN) of anther development that mainly consists of transcription factors (TFs) and their regulated target genes. In addition, miRNAs have also been reported to be involved in plant flowering and the subsequent anther developmental pathways by repressing the transcriptional or translational activities of target genes in GRNs [8,9,10]. Therefore, gene regulatory models in anther development mainly include two types: (1) *trans*-acting factors that interact with *cis*-regulatory elements, and (2) miRNA-mediated repression of target gene expression.

The intrinsic content of a GRN is the interactions among proteins, RNAs, and DNA motifs in the cell. Recently, the “ceRNA hypothesis” has been proposed as a novel type of gene regulatory model based on interactions between RNAs [11]. As endogenously transcribed RNAs, ceRNAs could function to absorb matching miRNAs like sponges (thus ceRNAs could be named as miRNA sponges as well) via miRNA response elements (MREs) and ultimately de-repress the transcriptional or/and translational limitations acting on miRNA target genes. A ceRNA MRE interacts with its corresponding mature miRNA sequence via an incompletely complementary manner, different from the interaction at target sites between miRNAs and their target genes. Initially, miRNA sponges were designed as artificial molecules to inhibit the functions of a corresponding miRNA family [12]. Meanwhile, an endogenous ”target mimicry”, originally annotated as a long noncoding RNA (lncRNA), *IPS1*, was first discovered and identified in *Arabidopsis*, and had an important function in regulating phosphate content by sponging *miR-399* that controlled the target gene *PHO2* [13]. This is a novel mode of post-transcriptional regulation, namely, miRNA (e.g., *miR-399*) transcription levels can be repressed by other RNA molecules (e.g., *IPS1*) and the expression of miRNA target gene (e.g., *PHO2*) is regulated. In subsequent studies, more ceRNAs originating from lncRNAs have been identified and found to be involved in the stress response and developmental processes in animals, and even play important roles in cancer [14,15,16].

Except lncRNAs, endogenous transcripts of pseudogenes, transposable elements (TEs), simple sequence repeats (SSRs), and circular RNAs (circRNAs), which are considered to be transcripts lacking protein-coding potential, have been reported to have ceRNA functions in vivo to control transcriptional levels of miRNAs and contribute to survival and development of diverse organisms [17,18,19,20]. Similarly, transcripts of protein-coding genes (coding genes) have also been found to have ceRNA functions [21]. In addition, a recent study from the evolutionary perspective supports the notion that ceRNA functions may be a common feature of pseudogenes [22]. These findings suggest that ceRNAs are important regulators in the normal developmental and stress-response processes of organisms. More importantly, it has been shown that nearly all types of relatively long transcripts in transcriptomes (e.g., pseudogenes, lncRNAs, TEs, and coding genes), but not small RNAs (rRNAs, tRNAs, snRNAs, and snoRNAs), have the possibility to function as ceRNAs [23,24,25]. 

Following the initial burst of ceRNA case studies, genome-wide identification and analysis of ceRNAs have been reported in plants and animals [26,27,28], and several databases of ceRNAs have been established based on experimental results and computational analyses [29,30,31]. These case studies and databases provide valuable information for revealing gene regulatory pathways relevant to ceRNA. Notably, a recent study on gastric cancer revealed a network-like ceRNA regulatory pathway including two ceRNAs, two miRNAs, and one target gene [16]. These results indicate that interactions among ceRNAs, their sponging miRNAs, and miRNA target genes in transcriptomes could comprise a ceRNA-miRNA-target gene regulatory network [23]. Several ceRNA-miRNA-target gene regulatory networks have been reconstructed in human cancers by using computational approaches, while bioinformatics-based studies on ceRNA profiles and their regulatory networks are still fewer in plants.

Several reported studies have described miRNA regulatory pathways in plant flower development or sexual reproduction [32,33,34,35]. Nevertheless, few studies have systematically introduced ceRNAs and their functions in the ceRNA-miRNA-target gene regulatory network controlling anther development. Although ceRNAs are novel players in post-transcriptional regulation, their potential roles in anther development are largely unknown. In this study, we identified and analyzed maize anther development-related ceRNAs by using RNA and small RNA transcriptome data in two maize inbred lines (lines L7 and L30) during three anther developmental stages (S8, S9, and S10). We then reconstructed ceRNA-miRNA-target gene regulatory networks in maize anther and found that many target genes and miRNA-target gene pairs in the networks participated in maize anther development based on the previous reports. These findings not only extend our understanding on maize anther GRNs by introducing ceRNA regulators, but also indicate that the ceRNA-miRNA-target gene regulatory networks may function in regulating anther development and sexual reproduction in plants.

## 2. Results

### 2.1. The Landscape of Transcribed Loci in Maize Anther Development

**Figure 1:**
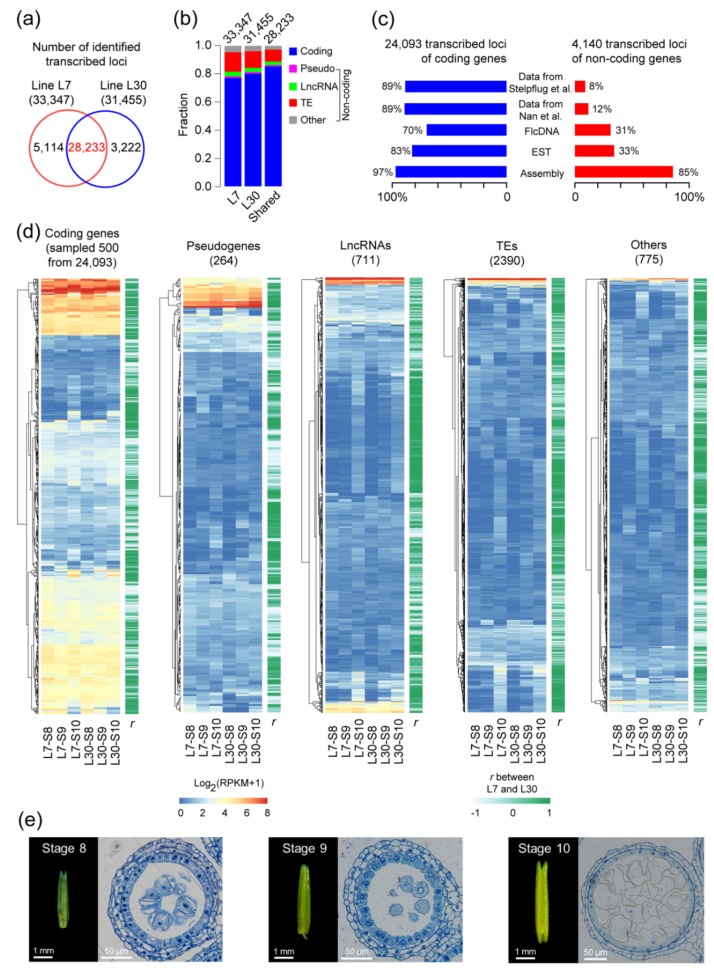
Identification and classification of transcribed loci in the developing maize anther. (**a**) Numbers of transcribed loci identified in transcriptomes of the two maize lines. Only the 28,233 loci that overlapped between the two lines (marked in red) were used in this study. (**b**) Classification of the transcribed loci into five types. “Coding” represents coding genes, and “Pseudo” represents pseudogenes. (**c**) Confirmation of the coding and non-coding transcribed loci by using the published data and the public databases. Maize anther transcriptome datasets from two previously-published studies were reanalyzed here [36,37]. (**d**) Transcription expression patterns of transcribed loci used in this study. Anther developmental stages 8, 9, and 10 are represented by S8, S9, and S10, respectively. Transformed transcription levels >8 were designated as 8 to facilitate the comparisons of transcription expression patterns among the five types of transcribed loci. Correlation of the transcription expression levels between maize lines L7 and L30 at the three stages for each transcribed locus was estimated using Pearson’s correlation coefficient (*r*). (**e**) Anther phenotypes (left) and transverse section analysis (right). We sequenced transcriptomes from maize anther at developmental stages of S8, S9, and S10, respectively. The developmental stages of anther samples were identified by transverse section analysis.

To perform genome-wide identification of ceRNAs and investigate their potential functions in maize anther development, transcribed loci should first be identified in transcriptomes. We constructed and sequenced RNA-sequencing (RNA-seq) libraries and their corresponding small RNA-seq libraries by using anther tissues sampled at three developmental stages (S8, meiosis stage; S9, microspore stage; S10, vacuolated microspore stage) (Figure 1). To identify stably transcribed loci and construct conserved gene regulatory networks in developing maize anther, transcriptomes of two maize lines with Oh43 genetic background, lines L7 (6 samples) and L30 (5 samples), were sequenced respectively and only their shared transcribed loci were used in the analyses (Table S1). When identifying transcribed loci in each line, we chose two thresholds including (1) the transcribed loci should have relatively high transcription levels, and (2) their transcripts should have intact sequences. By mapping the RNA-seq transcriptome data obtained above to the maize B73 reference genome sequence (version AGPv4), we identified 142,328 transcripts corresponding to 50,342 transcribed loci, with an average of 2.83 transcripts per transcribed locus. In the following analysis, the transcribed loci with transcriptional levels >0.5, namely, read counts per kilobase per million mapped reads (RPKM) >0.5, and transcribed lengths >200 nt, were used to avoid transcript noise in the RNA-seq data, which resulted in 33,347 and 31,455 transcribed loci being selected from lines L7 and L30, respectively (Figure 1a). Based on gene models referring to public databases such as MaizeGDB, NCBI, and Ensembl, and computational annotation information produced in this study, the stably transcribed loci were annotated and divided into five types including coding genes, pseudogenes, lncRNAs, TEs, and other type (Figure 1b). Among these stably transcribed loci, coding genes were the predominant group (77.06–80.17%) when compared with the non-coding loci (pseudogenes, 1.10–1.20%, lncRNAs, 3.05–3.36%, TEs, 11.82–13.86%, and other type, 3.87–4.52%). To further identify the conserved and significant functional interactions among ceRNAs, miRNAs, and target genes, the transcribed loci shared between lines L7 and L30 were selected for the next step analysis. As a result, 8,336 transcribed loci identified in only one of the two maize lines were filtered out, and the retained 28,233 transcribed loci were considered to be stably expressed in the two maize lines during three anther developmental stages (Figure 1a and Table S2).

Among the 28,233 transcribed loci, 24,093 loci were annotated as coding genes and the remaining 4,140 loci were annotated as non-coding genes. For the 24,093 coding-gene loci, nearly 90% were also detected in two RNA-seq datasets of maize anther reported previously [36,37], which were reanalyzed in this study (Figure 1c). Additionally, 69.83% and 82.64% of the coding-gene loci were repeatedly detected in the maize full-length cDNA (FlcDNA) and expressed sequence tag (EST) databases, respectively (Figure 1c). The high overlap rates indicate that the coding-gene transcripts identified here are really stably transcribed and have intact sequences.

Among the 4,140 non-coding transcribed loci, the relatively low rates, 7.80% and 11.76%, were repeatedly detected in the other two RNA-seq datasets, respectively (Figure 1c). The reason may be that the RNA-seq libraries for the other two datasets were constructed by using polyA-containing mRNAs which limited enrichment of non-coding transcripts [36,37]. In addition, 30.56% and 33.31% of the non-coding loci were repeatedly detected in the maize FlcDNA and EST databases respectively, while 85.26% of the non-coding loci were identified in the de novo assembled transcripts, which is similar to the overlap rate (97.08%) of the coding-gene loci (Figure 1c). Considering the majority (85.26%) of the non-coding transcribed loci and the moderate subsets (30.56% and 33.31%) confirmed in de novo assembled transcripts and the public databases respectively, the 4,140 non-coding loci can be used for ceRNA detection, and include 264 pseudogenes, 711 lncRNA loci, 2,390 TEs, and 775 unannotated RNA loci (Figure 1d and Table S2).

The transcriptional landscapes are diverse among the different types of transcribed loci in maize anthers (Figure 1d). In general, transcriptional levels of coding genes were higher than those of the non-coding loci. Notably, a larger proportion of pseudogenes were more highly transcribed than the other types of non-coding loci. Moreover, the expression patterns of a majority of transcribed loci were similar between lines L7 and L30 during the three developmental stages. On average, 61.55% of the transcribed loci showed positive correlations at transcriptional levels (Pearson’s correlation coefficient, *r* > 0.3) between the two lines during the three stages, and 40.32% of the transcribed loci were highly associated (*r* > 0.8) (Figure 1d). This finding further reinforces our conclusion that the majority of the transcribed loci identified here is not only stably expressed, but also show similar expression patterns in the different lines.

Taken together, comparative analyses among the published transcriptomes, the public FlcDNA and EST databases, and the anther transcriptomes sequenced here suggest that the 28,233 transcribed loci identified in this study are creditable and can be used for the subsequent analysis.

### 2.2. The microRNA (miRNA) Profile in Maize Anther Development

**Figure 2:**
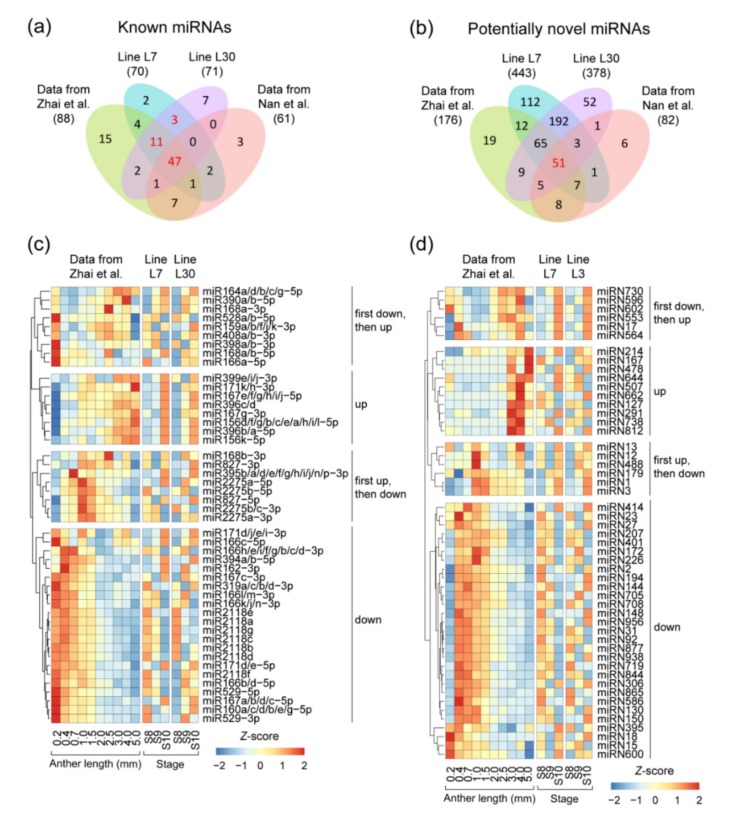
Identification of transcribed microRNAs (miRNAs) in the developing maize anther. Numbers of identified (**a**) known and (**b**) potentially novel miRNAs were shown. The maize anther small RNA transcriptome data from two previous studies was reanalyzed here [37,38]. Sixty-one known miRNA groups and 51 potentially novel miRNA groups were used in the following analysis (marked in red). Transcription expression patterns of identified (**c**) 47 known and (**d**) 51 potentially novel miRNAs were categorized into four classes based on the reanalyzed results of Zhai et al.’s data with 10 anther developmental stage points [38]. Transcription expression levels of each miRNA group were normalized to *Z*-scores for data from Zhai et al., line L7 and line L30 separately.

miRNAs, as key regulatory nodes in ceRNA-miRNA-target gene regulatory networks, are regulated by their corresponding ceRNAs, and further serve as the regulators of their target genes. In this study, small RNA-seq data were generated from the same samples as sequencing of the long RNAs (Table S1 and Figure S1). Among all identified miRNAs, 321 miRNA loci were annotated in the maize reference genome according to miRBase (release 22) and corresponded to 203 unique miRNA groups after merging the miRNA loci with the same mature sequences. We then selected and used miRNAs with read counts per million mapped reads (CPM) values >1 in at least one sample in each of the two lines. Based on this selection condition and the known miRNA annotation, 70 and 71 miRNA groups were identified in small RNA transcriptomes of lines L7 and L30 respectively, with the proportion of ~35% of the known maize miRNA groups (70/203 and 71/203) (Figure 2a). The known miRNAs not identified here may be highly expressed at other anther developmental stages or in other maize tissues. Among these identified known miRNAs, 61 miRNA groups were shared between lines L7 and L30, and 47 were confirmed in both the two published maize anther small RNA-seq datasets [37,38], which were reanalyzed by using the same method used in this study (Figure 2a). This finding indicates that the known miRNA groups identified and selected here are stably transcribed during anther development in maize. Therefore, all the 61 known miRNA groups are creditable, and can be used in the following analysis (Table S3).

Except for the known miRNA groups, we also identified potentially novel miRNA groups from the small RNA-seq data as determined by four thresholds including (1) a relatively high expression level (CPM > 1), (2) a meaningful secondary structure (*p* < 0.05), (3) shared between the two lines, and (4) further confirmed in the other two published datasets [37,38]. After being screened, 51 potentially novel miRNA groups were selected, confirmed, and used in the following analysis (Figure 2b and Table S4). None of the 51 potentially novel miRNA groups were found to have the same mature sequence with plant known miRNAs (miRBase, release 22), indicating that the novel miRNA groups identified here may be maize anther-specific miRNAs.

Based on the small RNA dataset of maize anther published previously [38], the expression patterns of the 47 known miRNAs shared among the four miRNA datasets can be divided into four types including (1) eight up-regulated miRNAs, (2) 22 down-regulated miRNAs, (3) eight miRNAs with first up-regulation and then down-regulation, and (4) nine miRNAs with first down-regulation and then up-regulation (Figure 2c). Similarly, the 51 potentially novel miRNAs can also be clustered into the same four types (Figure 2d).

### 2.3. Competing Endogenous RNAs (ceRNAs) and Target Genes of miRNAs

We predicted ceRNAs and target genes of the miRNAs selected above using computational approaches. Among the 28,233 transcribed loci, 583 (2.06%) and 857 (3.04%) loci were predicted as ceRNAs and target genes of miRNAs, respectively (Table S5). Thirty-one loci (0.11%) were predicted as both ceRNAs and target genes of miRNAs, which is significantly higher than the expected value (0.06% of 28,233 loci, 17 loci; one-tailed Fisher’s exact test, *p* = 0.0297). The number of predicted ceRNAs (3.92) per known miRNA is significantly smaller than that (12.42) per potentially novel miRNA. Similarly, the average number of predicted target genes (7.18) per the known miRNA is relatively lower than that (10.87) per the potentially novel miRNA (Table S5).

**Figure 3:**
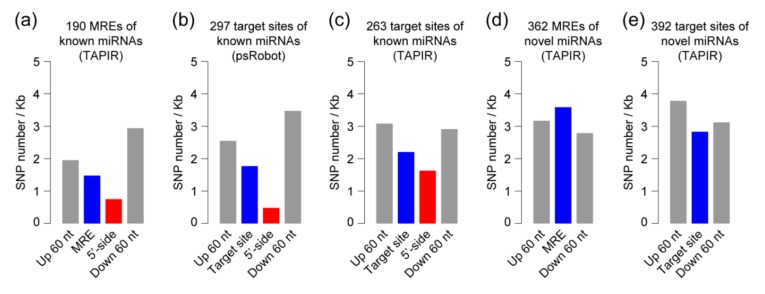
Sequence conservation of miRNA response elements (MREs) and target sites of known and potentially novel miRNAs. Single nucleotide polymorphism (SNP) density distribution along the miRNA binding sites and the flanking regions (up- and down-stream 60 nt, respectively) in (**a**) competing endogenous RNAs (ceRNAs) of known miRNAs, (**b**) target genes of known miRNAs by psRobot software and (**c**) by TAPIR program. SNP density distribution along the miRNA binding sites and the flanking regions in (**d**) ceRNAs of potentially novel miRNAs and (**e**) target genes of potentially novel miRNAs by TAPIR program. There were four predicted target genes by using the psRobot program for potentially novel miRNAs, but no SNP site existed at the four target sites and their flanking regions. “MRE and target site” indicate binding regions recognized by mature miRNA sequences on predicted ceRNA and target genes respectively, and are marked in blue. “5′-side” represents binding regions recognized by the 2nd to 7th nucleotides from the 5′ end of miRNA mature sequences (marked in red).

Although we carefully filtered the ceRNAs and target genes by using well-developed and commonly used programs [23,39,40,41], it is still uncertain whether these predicted pairs of ceRNA-miRNA and miRNA-target gene could actually interact with each other. In general, functional elements are often conserved in genomic sequences in different individuals or species because of their functional constraints. Some miRNAs as well as their target sites are conserved in sequences among closely related species, even across land plants [42,43]. Therefore, we propose that the MRE sequences of ceRNAs should be conserved if the ceRNAs computationally identified here have genuine functions in regulating their downstream miRNAs. To verify this hypothesis, we first identified 0.34 million high-quality single nucleotide polymorphism (SNP) sites by using the transcriptome data of the 11 anther-tissue samples in lines L7 and L30. We then investigated SNP densities both at and around the predicted MRE region where a known mature miRNA sequence and its corresponding ceRNA fragment bind or interact. As a result, for the known miRNAs, we found that the SNP density in the MRE region is obviously lower than that in the MRE flanking regions represented by both the upstream and downstream 60 nt sequences (Figure 3a). In addition, the 5′-end miRNA mature sequence is found to be more important in recognizing the target gene or ceRNA when compared to the 3′-end miRNA mature sequence in plants [44,45]. Here, we found that MRE regions binding to the 5′-end sequence of known miRNAs (the 2nd to 7th nucleotides of mature miRNA, named as “5′-side” region in this study) has a much lower SNP density than the other investigated regions (Figure 3a), which is consistent with the functional significance of mature miRNA 5′-side region reported previously in plants [44,45]. The sequence conservation of MREs in predicted ceRNAs suggests that these transcripts most likely have ceRNA functions. On the other hand, the conservation of sequences was also observed at the target sites of known miRNAs predicted by the psRobot program. SNP density in the target regions interacting with the known miRNA 5′-side sequences is much lower than those in the flanking regions (Figure 3b), which is reliable evidence for the predicted target genes of known miRNAs. Moreover, the low SNP density at the target sites can also be observed in target genes predicted by the TAPIR program (Figure 3c). Notably, for the potentially novel miRNAs, SNP densities at interacting sites of ceRNAs and target genes were similar to those in flanking regions (Figure 3d,e). This result is consistent with the previous reports, suggesting that poorly conserved or young miRNAs may be selectively neutral, with limited selection pressure on them [46]. 

After investigating the functional characters of coding genes predicted as ceRNAs or target genes, we found that the proportion of coding gene-originated ceRNAs annotated as TF genes is significantly lower than that of coding gene-originated target genes annotated as TF genes (5.99% versus 13.44%; Fisher’s exact test, *p* = 0.0001). This difference was more significant for known miRNAs (6.38% versus 21.24%, *p* = 5.48 × 10^–5^), but was not significant for potentially novel miRNAs (5.75% versus 4.90%, *p* = 0.7051). This suggests that target genes likely tend to become key regulatory nodes in anther GRNs, especially for target genes of known miRNAs. The difference in the relative numbers of TFs between ceRNAs and target genes is also reflected in the biological function enrichment results, in which ceRNAs are mainly involved in metabolic and protein modification processes, while target genes are functionally enriched in flower development and cellular regulation processes (Figure S2). These results indicate that coding gene-originated ceRNAs and target genes may represent two groups of genes with different functions. In other words, coding gene-originated ceRNAs tend to encode proteins involved in common cellular functions such as catalytic proteins, while coding gene-originated target genes are biased toward regulation of tissue-specific biological processes and encode proteins such as TFs.

### 2.4. Reconstructing ceRNA-miRNA-Target Gene Regulatory Networks in the Developing Maize Anther

**Figure 4:**
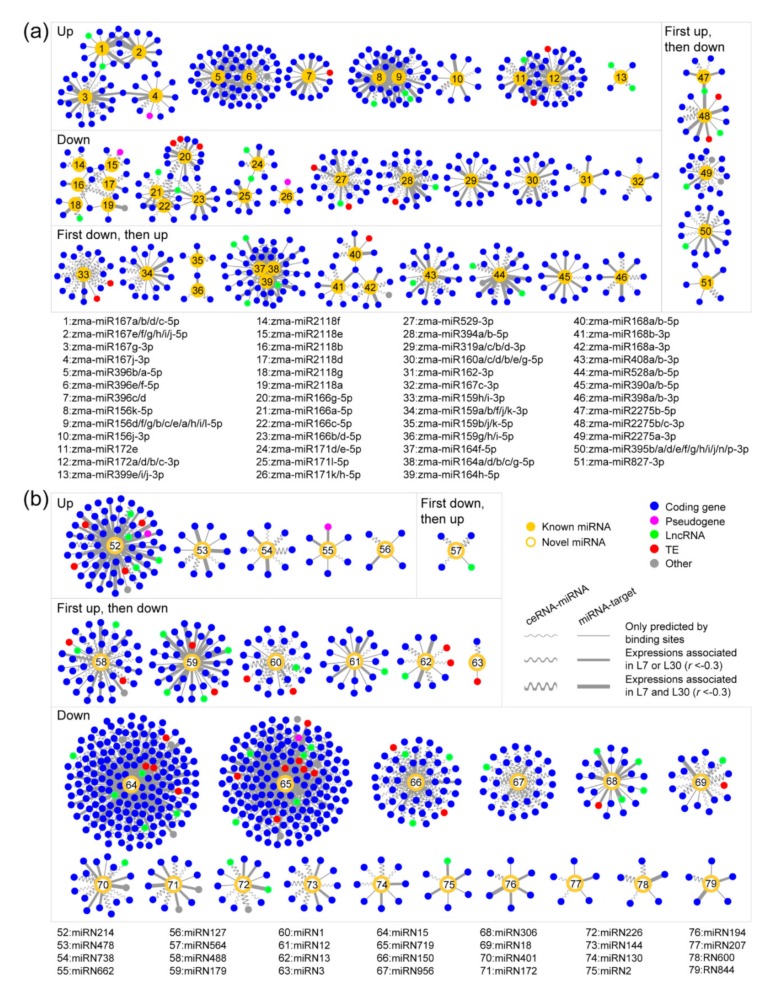
The 79 ceRNA-miRNA-target gene regulatory networks in the developing maize anther. There were (**a**) 51 known miRNA-mediated and (**b**) 28 potentially novel miRNA-mediated ceRNA-miRNA-target gene regulatory networks. The 51 known miRNA-mediated networks were first classified into four categories (i.e., “Up”, “Down”, “First up, then down”, and “First down, then up”) based on the expression patterns of known miRNAs shown in Figure 2, and then were numbered from “1” to “51”. The 28 potentially novel miRNA-mediated regulatory networks were also classified and numbered from “52” to “79”. Please refer to Table S5 for the detailed information of ceRNAs, miRNAs, and target genes located in the 79 regulatory networks.

**Figure 5:**
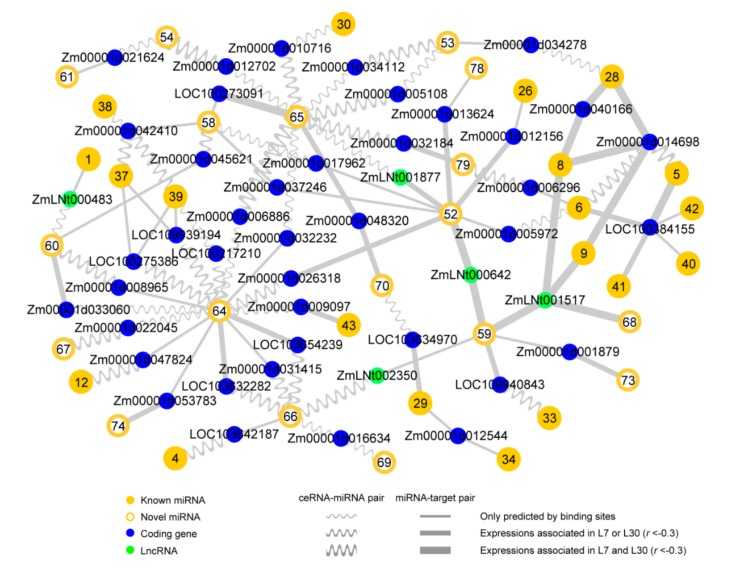
The complex crosstalk among the 38 reconstructed ceRNA-miRNA-target gene regulatory networks. Numbers in the yellow dots and circles represent 20 known miRNAs and 18 potentially novel miRNAs respectively, that are numbered in Figure 4.

After identifying miRNAs and stably transcribed loci in the maize anther, we computationally predicted ceRNA-miRNA and miRNA-target gene pairs and found that the interaction pairs displayed sequence conservation in miRNA binding regions (MREs and target sites). By integrating ceRNA-miRNA and miRNA-target interaction pairs and excluding miRNA-mediated pathways only containing ceRNAs or target genes, we reconstructed 79 ceRNA-miRNA-target gene regulatory networks during maize anther development (Figure 4 and Figure S3). These networks include 576 predicted ceRNAs, 79 miRNAs (51 known and 28 potentially novel miRNAs), and 753 target genes, which together comprise 619 ceRNA-miRNA pairs and 869 miRNA-target gene pairs. In addition, one gene (*Zm00001d012381*) was predicted as both a ceRNA and a target gene of a potentially novel miRNA (*zma-miRN15*) in the network. Meanwhile, there are complex links between 20 known miRNA- and 18 potentially novel miRNA-mediated regulatory networks (Figure 5), and relatively simple links exist among other six known miRNA- and one potentially novel miRNA-mediated regulatory networks (Figure S4).

It is well known that ceRNAs can negatively regulate the matched miRNAs at the level of transcription, and that miRNAs can repress the transcription levels of their target genes. Therefore, it can be assumed that transcriptional levels of predicted ceRNAs and target genes should be negatively correlated with those of their interacting miRNAs across the three anther developmental stages (S8 to S10). Through expression correlation analysis on ceRNA-miRNA and miRNA-target pairs, we found that 47.17% of ceRNA-miRNA pairs (292/619) showed negative correlations (*r* < −0.6) in at least one of the two maize lines. Moreover, 16.32% of ceRNA-miRNA pairs (101/619) were found to be negative correlations in both the two maize lines. For the miRNA-target gene pairs, 41.20% (358/869) and 17.49% (152/869) were observed to be negative correlations (*r* < –0.6) in at least one line and in both the two lines, respectively. These negatively associated pairs may represent more credible interactions in the reconstructed networks.

Among the 576 predicted ceRNAs, there are 520 coding genes, three pseudogenes, 24 lncRNAs, 21 TE genes, and eight other types. Compared with the initially identified levels in the 28,233 transcribed loci (Figure 1b), the proportions of coding genes (85.34% versus 90.28%) and lncRNAs (2.52% versus 4.17%) predicted as ceRNAs were significantly increased in the networks, while the proportions of the other transcript types predicted as ceRNAs were reduced. There were 682, 3, 36, 21, and 11 target genes annotated as coding genes, pseudogenes, lncRNAs, TE genes, and other types, respectively. Similarly, coding genes (85.34% versus 90.57%) and lncRNAs (2.52% versus 4.78%) were significantly enriched with target gene functions. Notably, the enrichment of lncRNAs predicted as ceRNAs and target genes were 1.65- and 1.90-fold respectively, which are higher than the enrichment of coding genes with both 1.06-fold. These results suggest that coding genes and lncRNAs have higher probabilities involving the ceRNA-miRNA-target gene regulatory networks, especially for lncRNAs.

### 2.5. The ceRNA-miRNA-Target Gene Regulatory Networks during Maize Anther Development

Having reconstructed the ceRNA-miRNA-target gene regulatory networks in the maize anther and revealed its general features, we further investigated whether these networks have significant functions in regulating maize anther development. One approach for verifying their functional significance is to investigate functions of the miRNA-target genes identified in these networks. Specifically, three methods were used in this study: (1) performing gene ontology (GO) analysis for the target genes, (2) determining whether some of the target genes or their orthologs are the reported functional genes regulating anther development, and (3) comparing the overlap between the miRNA-target gene pairs reported previously and identified here. The GO analysis showed that the target genes are functionally enriched in flower development genes (Figure S2). Permutation analysis using randomly selected transcripts with the same sample size as target genes (10,000 times) indicates that the enrichment result is not attributed to the sample bias that transcribed loci were identified from transcriptomes of anther tissues (*p* = 0.0164). This finding roughly supports our hypothesis that the reconstructed ceRNA-miRNA-target gene regulatory networks may contribute to maize anther development. Nevertheless, a more detailed analysis should be performed to further verify our hypothesis. A maize GMS gene *ZmMs7* reported previously in our laboratory [47] was predicted as the target gene of a potentially novel maize miRNA, *zma-miRN15*, in this analysis. Additionally, in our recent review, we identified 79 GMS orthologous genes in maize based on the GMS gene information reported in *Arabidopsis*, rice, and other plant species [4]. The functional roles of these GMS genes have been well studied, and found to be involved in crucial transcription, lipid metabolism, polysaccharide metabolism, and other processes, which play important roles in maize anther development. In this study, 86.08% (68/79) of these GMS orthologous genes were identified as transcribed loci, and six of them were located in the reconstructed regulatory networks as target genes. Five genes (*Zm00001d012544*, *Zm00001d043131*, *Zm00001d002929*, *Zm00001d034701*, and *Zm00001d046537*) may be regulated by known miRNAs, and *Zm00001d053895* was predicted to be the target gene of a potentially novel miRNA. The relative enrichment was 2-fold for the six genes (6/1177; *p* = 0.0599) and increased to 3-fold for the five genes targeted by known miRNA (5/525, *p* = 0.0198) when compared with the background (68/24,093). In addition, many reported miRNA-target gene pairs related to flower or anther development were found to be included in the reconstructed regulatory networks, such as *miR156*-*SPL* (*SQUAMOSA PROMOTER BINDING PROTEIN-LIKE*), *miR159*-*MYB*, *miR160*-*ARF* (*AUXIN RESPONSE FACTOR*), *miR164*-*NAC* (*NAM*, *ATAF*, and *CUC*), *miR172*-*AP2* (*APETALA2*), and *miR319*-*TCP* (*TEOSINTE BRANCHED1*, *CYCLOIDEA*, and *PCF*) pairs [10]. These results from the three aspect analyses indicate that the reconstructed ceRNA-miRNA-target gene regulatory networks are likely involved in the process of maize anther development.

**Figure 6:**
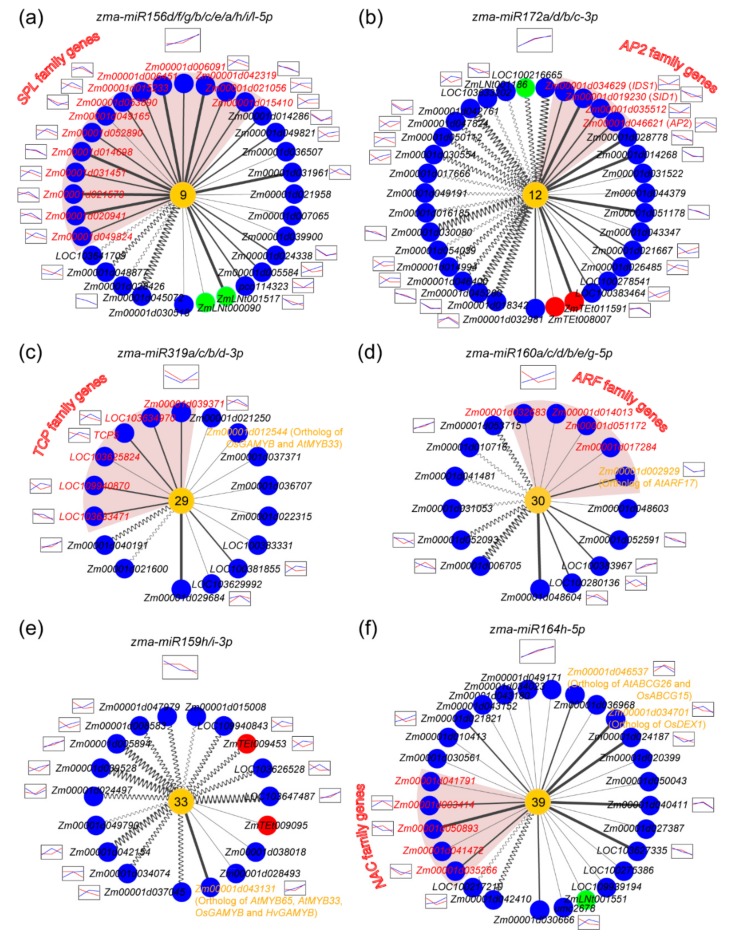
The six known miRNA-mediated regulatory networks functionally associated with maize anther development. The six networks mediated by the known miRNAs of (**a**) *zma-miR156*, (**b**) *zma-miR172*, (**c**) *zma-miR319*, (**d**) *zma-miR160*, (**e**) *zma-miR159*, and (**f**) *zma-miR164* are shown. The transcription expression patterns of corresponding miRNAs are shown at the top of the networks based on small RNA transcriptomes in maize lines L7 (marked in red curve) and L30 (marked in blue curve). The transcription expression patterns of ceRNA or target genes that were negatively associated with miRNA expression in at least one line are shown beside them. The names and the backgrounds of target genes in the same gene family in each network are marked in red. The names of genes with orthologs that participate in anther development in other plant species are shown in yellow. Other legend of this figure is the same as that of Figure 4 and Figure 5.

*SPL* family genes encode a group of plant-specific transcription factors participating in flower development [48,49]. In addition, *SPL* genes are the targets of *miR156*. There are 27 target genes in the reconstructed *zma-miRNA156*-mediated network, of which 14 belong to the *SPL* family. The expression levels of 13 *SPL* genes were negatively correlated (*r* < –0.3) with that of *zma-miR156* in at least one line (Figure 6a). Notably, among four ceRNAs located in the *zma-miRNA156*-mediated network, *Zm00001d045072* (a gene with unknown function) and *Zm00001d048877* (a leucine-rich repeat transmembrane protein kinase gene) were found to be negatively associated with *zma-miR156* at expression levels.

In the *zma-miR172*-mediated regulatory network, there are 17 ceRNAs and 17 target genes (Figure 6b). Expression levels of 12 ceRNAs and 11 targets were negatively associated with that of *zma-miR172*. It was reported that *miR172* is crucial for plant sexual reproduction [50]. Z*ma-miR172e*, one of the *zma-miR172* family members, has essential roles in stamen identity by targeting *AP2* and two *AP2*-like genes (*IDS1* and *SID1*) in maize [9,51]. In this study, the expression levels of *AP2* family genes including *SID1* and *AP2* were negatively correlated with that of *miRNA172* (Figure 6b). Interestingly, *miR156*-target genes can regulate *miR172* expression [52], which indicates that the regulatory networks underlying flower or anther development may be complicated since links exist among different miRNA-mediated regulatory networks. The results obtained here indicate that the *zma-miR172*-mediated regulatory network not only participates in maize floral identity, but also may be involved in the development of anther and tassel.

In the *zma-miR319*-mediated regulatory network, six of 15 target genes belong to the *TCP* family (Figure 6c). The *miR319*-regulated *TCP* genes are considered to negatively regulate secondary wall-thickening in floral organs, overexpression of *TCP24* causes male sterility, and the *miR319* loss-of-function mutant exhibits abnormal stamens in *Arabidopsis* [53,54]. These reports suggest that *miR319* and its target *TCP* genes play important roles in anther development. More importantly, we found that *Zm00001d012544*, maize ortholog of *OsGAMYB*, was one of the predicted target genes of *zma-miR319* (Figure 6c). *OsGAMYB* has essential functions in floral organ and pollen development in rice [55]. Additionally, *Zm00001d012544* is highly expressed in the maize anther (information from MaizeGDB). Therefore, *Zm00001d012544* may be an important regulator of maize anther development.

*ARF* family genes are the targets of *miR160*. We identified five *ARF* genes in the *zma-miR160*-mediated network (Figure 6d). Among them, *Zm00001d002929*, ortholog of *AtARF17*, was negatively associated with *zma-miR160* expression and highly expressed in the maize anther (information from MaizeGDB). Interestingly, *AtARF17* was reported to participate in pollen wall formation in *Arabidopsis*, and its loss-of-function results in male sterility [56]. Therefore, *Zm00001d002929* may be an important regulator of maize reproduction. In addition, there are six predicted ceRNAs for *zma-miR160*, three of which have expressed associations with *zma-miR160*. These results indicate that *miR160*, combined with its ceRNAs and *ARF* targets, may play a crucial role in regulating maize anther development.

In two other networks, *zma-miR159* targeted *Zm00001d043131* (ortholog of *OsGAMYB*), and *zma-miR164* targeted *Zm00001d034701* (ortholog of *OsDEX1*) and *Zm00001d046537* (ortholog of *AtABCG26* and *OsABCG15*) (Figure 6e,f). *OsGAMYB*, *OsDEX1*, and *OsABCG15* are key functional genes in rice anther development and play important roles in male fertility [55,57,58]. Five *NAC* family genes were predicted to be target genes of *zma-miR164*, and expression levels of three of them were negatively correlated with that of *zma-miR164* (Figure 6f). Although miRNA paralogs belonging to the same miRNA family were slightly different in terms of numbers and types of their predicted ceRNAs and target genes, their regulatory network were relatively stable, and the majority of functional genes were shared between miRNA paralogs (Figure S5). 

Taken together, we can conclude that the reconstructed ceRNA-miRNA-target gene regulatory networks likely contribute to maize anther development.

### 2.6. Novel miRNAs Integrated in the ceRNA-miRNA-Target Gene Regulatory Networks

**Figure 7:**
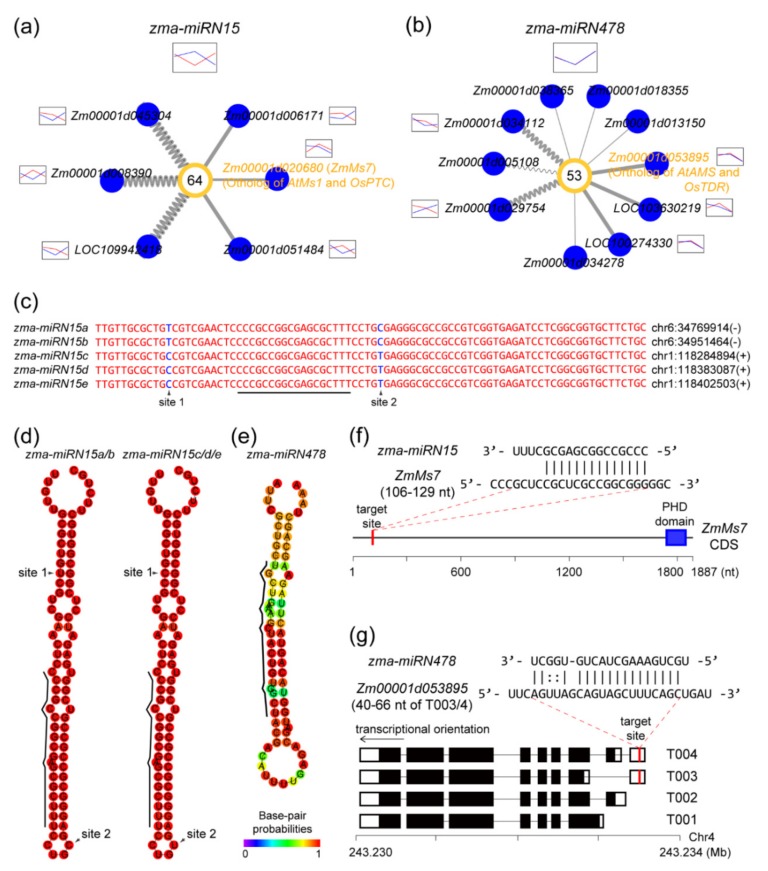
The two potentially novel miRNA-mediated regulatory networks involved in maize anther development. Networks mediated by the two potentially novel miRNAs of (**a**) *zma-miRN15* and (**b**) *zma-miRN478* are shown. For the *zma-miRN15*-mediated network, there were actually 80 ceRNAs and 98 targets (Figure 4b and Table S5). Legend in Figure 7a,b was the same as that of Figure 4, Figure 5, and Figure 6. (**c**) Genomic sequences of *zma-miRN15* precursors at five loci (*zma-miRN15a*, *b*, *c*, *d,* and *e*). Genomic position of the first nucleotide at the 5′ end of each locus is presented at the right side of the sequence. Region of *zma-miRN15* mature sequence is presented by the black line. Nucleotides at two sites (“site 1” and “site 2”) are marked in blue. (**d**) Stem-loop structures of two groups of *zma-miRN15* precursors (*zma-miRN15a*/*b* and *zma-miRN15c*/*d*/*e*) and (**e**) of *zma-miRN478* precursor. Region of *zma-miRN15* and *zma-miRN478* mature sequences are represented by the black line at the left side. (**f**) Target site of *zma-miRN15* on *ZmMs7* coding sequence. (**g**) Two target sites of *zma-miRN478* on two isoforms (T003 and T004) of *Zm00001d053895*. Black and white boxes indicate translated and untranslated regions in the transcripts, respectively.

Target genes of potentially novel miRNAs were also found to be associated with anther development (Figure 7a, b). For instance, we found that *ZmMs7* is targeted by a potentially novel miRNA, *zma-miRN15* (Figure 7a). Recently, we reported that *ZmMs7* encoding a PHD-finger TF is crucial for anther development and male fertility in maize [47]. *ZmMs7* is the ortholog of *AtMS1* in *Arabidopsis* and *OsPTC1* in rice [59,60]. There were five miRNA homogenous loci (*zma-miRN15a*, *b*, *c*, *d*, and *e*) for *zma-miRN15* in maize genome, which could be classified into two types based on two nucleotide sites (T/C and C/T) (Figure 7c). Precursor sequences of the two type *zma-miRN15* could be predicted to form canonical stem-loop structures by computational analysis (Figure 7d). Except for four nucleotides at *zma-miRN15* 3′-end, the other 14 nucleotides were perfectly complemented with the predicted *ZmMs7* target sites (Figure 7f).

Another potentially novel miRNA, *zma-miRN478*, was found to target *Zm00001d053895*, maize ortholog of *AtAMS* in *Arabidopsis,* and *OsTDR* in rice. *AtAMS and OsTDR* are essential for anther development and male fertility [61,62]. Genomic sequences around *zma-miRN478* could be predicted to a stem-loop structure (Figure 7e). Notably, target sites of *zma-miRN478* were observed in two of the four annotated maize isoforms of *Zm00001d053895* (Figure 7g), indicating that maize-specific or younger miRNAs possibly participate in the ceRNA-miRNA-target gene regulatory network.

## 3. Discussion

### 3.1. The ceRNA Components in Transcriptomes

The number of transcripts from non-coding genes (e.g., pseudogenes, lncRNA loci, and TEs) is much smaller than that from coding genes. Consequently, the majority of predicted ceRNAs belong to coding genes. This finding indicates that the interaction between miRNAs and transcripts of coding genes in the transcriptomes may be more frequent than that was previously thought. It is possible that transcripts of some coding genes function, either fully or partly, in regulating miRNA activities via a ceRNA regulatory mechanism, while the functional roles of their coding protein products may be less important in some developmental stage(s) or tissue(s). This may be one reason to explain why the inconsistencies exist between the levels of transcribed RNAs and translated proteins for some protein-coding genes [63].

In addition, we found that lncRNAs were relatively enriched in ceRNAs when compared with other types of non-coding transcripts, which implies that lncRNAs may be the other major source of ceRNAs. Considering that the first plant ceRNA molecule was discovered in phosphate starvation status, and that in the subsequent reports a large percentage of ceRNAs were mainly identified under abnormal conditions such as stress-induced organisms, cancer, or tumor tissues [13,16,17], it can be inferred that the ceRNA regulatory pathway may be more active under stress conditions and be considered as a supplementary gene regulatory mechanism at the post-transcriptional level. In this study, we focused on the normal developmental process of maize anther, in which the ceRNA transcripts, especially for the non-coding transcripts, may be less active in the reconstructed regulatory networks. The initial amounts of different types of transcripts and the possible stress-activated mechanism of the ceRNA-miRNA-target gene regulatory network may account for the enrichment of coding gene- and lncRNA-originated ceRNAs in this study. Further studies are needed to address the ceRNA components in transcriptomes under both normal and stress-response conditions. 

### 3.2. ceRNAs Differ from Target Genes of miRNAs

Although ceRNAs and target genes of known miRNAs were predicted by a computational approach, it has been demonstrated that the complementary sites between ceRNAs and miRNAs, as well as between miRNAs and their target genes, are relatively conserved in sequence when compared with their flanking regions. In general, the sequence conservation in the genomic elements may indicate their functional consistence and significance. Therefore, the predicted ceRNAs and target genes of miRNAs could be used to reconstruct the ceRNA-miRNA-target gene regulatory networks. Before reconstructing the regulatory networks, we investigated their functional features. The ceRNAs represent the up-stream regulators that can repress the activities of their corresponding miRNAs, while the target genes can be considered as the down-stream effectors whose transcriptional or translational activities are repressed by the miRNAs. Although there are an increasing number of studies on ceRNA identification in plants and animals, these reports rarely address whether the general features of ceRNAs are different from other transcripts. It is well known that most target genes of conserved miRNAs are TF-coding genes involved in controlling developmental processes [46]. Here, we also found that the functions of proteins encoded by target genes tend to be TFs in the GRN, with their functions significantly enriched in flower development and cellular regulation. However, the proportion of TF-coding genes annotated as ceRNAs is similar to that of the whole background in the maize genome, and the ceRNAs mainly take part in biological functions such as metabolism and catabolic processes, which significantly differ from the functional roles of the TF-coding genes predicted as target genes. These results indicate that the different positions of ceRNAs and target genes in the ceRNA-miRNA-target gene regulatory network may be tightly associated with their different functional features.

### 3.3. The Functional Significance of the ceRNA-miRNA-Target Gene Regulatory Networks during Maize Anther Development

In maize, the proportion of tissue-specific transcribed genes in anther is the highest (6.0%) when compared with other tissues during various developmental stages (Figure S6), suggesting that maize anther transcriptome has a special content and that the GRN underlying anther development may be more complicated. Previous studies have identified a number of functional genes involved in anther development, including TF-coding genes that are crucial regulatory nodes in the networks, enzyme-coding genes that participate in lipid and carbohydrate metabolism, and transporter genes [2,5,6,7,64,65]. Additionally, several miRNAs combined with their target genes were found to have important functions in regulating the floral organ identity and the subsequent development of anther and pollen [8,9]. These protein-coding genes and miRNA regulators comprise a meaningful GRN framework for further studies on mechanisms of anther development in plants. Here, we further introduced ceRNAs into the GRN framework, and found that miRNA-target genes indirectly regulated by ceRNAs are functionally significant in anther development and male fertility. De novo genes and other newly originated genes including miRNAs are crucial genomic elements for functional novelty, and are important components comprising the functional pathway and GRN of organisms [66,67]. Among the maize GMS genes reported [4,47,68,69,70,71], *ZmMs7* was found to likely be the target of a potentially novel miRNA. These results not only extend the GRN of anther development by adding a new type of regulatory relationship (ceRNA-miRNA pair), but also reveal interactions among transcripts during anther development. At present, our understanding on the mechanisms of anther development and the ceRNA-miRNA-target gene regulatory network is far from complete. Here, all the identified ceRNAs, miRNAs, and target genes, along with their interaction relationships, provide a meaningful dataset for future studies. Therefore, more experimental studies and computational analyses should be performed to deeply understand the biological processes involved in anther development and male fertility in plants.

## 4. Materials and Methods

### 4.1. Plant Materials, Anther Samples, and Transcriptome Sequencing

The plants were grown in the field of the University of Sciences and Technology Beijing (USTB) with normal field management. Anther collection was performed as previously described [72]. Anthers with 2.5–4.0 mm length were collected from upper florets with two pairs of sharp forceps directly into microcentrifuge tubes, immediately frozen in liquid nitrogen and stored at –80 °C until use. Fifty to sixty anthers were collected for each biological replicate. At the same time, five fresh anthers were immersed in FAA solution (Coolabor, Beijing, China) overnight for transverse section SEM (scanning electron microscope) analyses. According to the results of SEM analyses, we ensured that the anthers collected were at the correct developmental stages (Figure 1e).

Total RNA was extracted from anther tissues using an RNAprep Pure Plant Kit (Polysaccharides and Polyphenolics-rich) (TIANGEN, Beijing, China). The small RNA-seq libraries were generated using NEBNext^®^ Multiplex Small RNA Library Prep Set for Illumina^®^ (NEB, USA) following the manufacturer’s recommendations. After 3′ and 5′ adapter ligation and cDNA synthesis, DNA fragments corresponding to 140–160 bp (the length of small RNA between 18–30 nt plus the 3′ and 5′ adaptors) were recovered by 8% PAGE. The library preparations were sequenced on an Illumina Hiseq 2500 platform and 50 bp single-end reads were generated. The ribosomal RNA was removed from the extracted RNA by Epicentre Ribo-zero™ rRNA Removal Kit (Epicentre, USA). RNA-seq libraries were constructed using the rRNA-depleted RNA by NEBNext^®^ Ultra™ Directional RNA Library Prep Kit for Illumina^®^ (NEB, USA) following manufacturer’s recommendations. After adapter ligation and cDNA synthesis, the library preparations were sequenced on an Illumina Hiseq 4000 platform and 150 bp paired-end reads were generated.

### 4.2. Analyses of Transcribed Loci and Single Nucleotide Polymorphism (SNP) in the Maize Transcriptomes

The ribosomal RNA-depleted RNA-seq libraries were constructed and sequenced from 11 anther tissue samples at three developmental stages (stages 8, 9, and 10) in two maize lines (L7 and L30) (Table S1). Raw sequencing reads were processed to remove adapter sequences, low quality reads (quality scores <20), and un-paired reads using NGSQCToolkit (version 2.3.3) [73]. The remaining high-quality clean reads were mapped to the maize B73 reference genome (version AGPv4, downloaded from Ensembl release 37) using TopHat2 with the default parameters [74]. Transcribed loci were identified in each sample using the cufflinks program without setting reference transcript annotations, and the results were then merged using cuffmerge [75]. Expression levels of the transcribed loci were estimated with the edgeR package using RPKM values [76]. The transcribed loci with RPKM values >0.5 and the lengths of their longest transcripts >200 nt in at least one sample of each line were used in the subsequent analyses. 

SNP sites in the two lines were identified via the alignments of the RNA-seq data in each sample using the Genome Analysis Toolkit (GATK) pipeline [77]. High-quality SNP sites (parameters used in GATK: QD > 2.0, FS < 60.0, MQ > 40.0, MQRankSum > –12.5, and ReadPosRankSum > –8.0) from these samples were used for further analysis. If at least three samples from one line were polymorphic at a site, this site is defined as an SNP site in the investigated line. Subsequently, SNP sites from the two lines were merged together to estimate the sequence conservation of MREs (miRNA matched regions by ceRNAs) and target sites (target gene matched regions by miRNA) located in ceRNAs and target genes, respectively. The sequence conservation levels were estimated by comparing SNP densities (the number of SNP sites per kilobase) between miRNA binding regions (MREs and target sites) and their flanking regions.

Short reads in the transcriptome data were assembled de novo into longer transcripts using the Trinity program with the default parameters [78,79]. The maize public FlcDNA and EST data were downloaded from The Maize Full-Length cDNA Project (http://www.maizecdna.org/), which contains 27,455 FlcDNA sequences and 364,385 EST sequences. The identified transcripts in this study were aligned to the FlcDNA and EST sequences using BLASTn [80]. The transcripts with similarities >85% and lengths >200 nt in the aligned regions were considered with expression evidences in FlcDNA or/and EST database. The two sets of RNA-seq data from maize anther tissues published previously were reanalyzed to confirm the credibility of the transcribed loci identified in this study [36,37].

### 4.3. Annotation and Classification of Transcribed Loci

After identifying the transcribed loci from the transcriptomes of maize anther, we annotated and classified these transcripts into five types including coding genes, pseudogenes, lncRNA, TEs, and other type. Gene model and annotation information based on the maize reference genome sequence (version AGPv4) were used to clarify the transcribed loci. Coding-gene models from MaizeGDB (Zm00001d) and the National Center for Biotechnology Information (B73_RefGen_v4) were merged together in this analysis. When there were overlapping gene-models present in the two datasets, those from MaizeGDB were shown. LncRNAs identified in four previous studies were merged into a single list representing the maize lncRNA models [81,82,83,84]. Additionally, lncRNAs annotated by Ensembl (release 39) and NCBI (B73_RefGen_v4) were added to the lncRNA models. Pseudogene models were predicted using pseudopipe based on coding-gene models [85]. TE annotation was downloaded from MaizeGDB (TransposableElements.gff3). When a transcribed locus overlapped with other transcript types, it was assigned to a certain transcript type according to the prior order of coding gene, pseudogene, lncRNA, and TE, and transcribed loci not assigned to any of the four transcript types were marked as “other type”. Transcribed locus IDs of coding genes were extracted from the MaizeGDB and NCBI databases, while transcribed locus IDs of pseudogenes, lncRNAs, TEs, or other type were designated ZmPGtxxxxxx, ZmLNtxxxxxx, ZmTEtxxxxxx, and ZmOTtxxxxxx, respectively (“xxxxxx” is a unique number specified for each locus, Table S2). TF annotation information of maize genes was obtained from the Plant Transcription Factor Database (http://planttfdb.cbi.pku.edu.cn/).

### 4.4. Analysis of Small RNA-Seq Data

Small RNA-seq data were initially processed using cutadapt 1.17 to remove adaptor sequences from the reads (https://cutadapt.readthedocs.io/en/stable/#). Reads containing ambiguous bases (marked as N), poly A tracts (length > 11; in miRBase, the longest poly A tract in a known mature miRNA sequence is 11 nt) and reads <18 nt or >25 nt were filtered out. The remaining reads were annotated using the Infernal package (version 1.1.2) based on the Rfam database (release 14.0) [86,87,88]. Reads similar to rRNAs, tRNAs, snRNAs, and snoRNAs were discarded. An investigation of the read length distribution was performed to confirm that the majority of the remaining reads were mature miRNA sequences. The retained high-quality reads were then mapped to the maize reference genome (AGPv4) using Bowtie software [89]. The number of reads mapped to known miRNA sequences were counted by Rsubread based on the maize miRNA models in miRBase (release 22) [90]. The MiRDeep2 package was used to identify potentially novel miRNAs transcribed in the maize anther [91]. The resulting predictions with significant randfold *p* values <0.05 and miRDeep2 scores >5 were considered as novel miRNA candidates. The potentially novel miRNAs were named miRNxxxx, in which xxxx is a unique number specified for each novel miRNA candidate. The transcription levels of the identified known and potentially novel miRNAs were estimated using edgeR based on the read counts that were normalized into CPM (read counts per million mapped reads) values. Known and potentially novel miRNAs with CPM values >1 in at least one sample were used in the subsequent analysis. Small RNA-seq data published previously in two independent studies were reanalyzed here using the method described above to confirm the credibility of identified miRNAs in this study [37,38]. Secondary structures of *zma-miRN15* and *zma-miRN478* precursors were re-predicted in the ViennaRNA Web Services (http://rna.tbi.univie.ac.at/).

### 4.5. Prediction of ceRNAs and Target Genes of miRNAs

ceRNAs and miRNA targets were predicted by comparing mature maize miRNA sequences and identified transcripts. Mature sequences of known maize miRNAs were obtained from miRBase, and mature sequences of maize potentially novel miRNAs were extracted from the miRDeep2 analysis results. The sequences of all isoforms of coding genes, pseudogenes, lncRNAs, TEs, and other type transcribed loci were extracted from the reference genome sequence based on their transcript models. The miRNA target genes were predicted using psRobot v1.2 [39] and TAPIR v1.2 [40], respectively. Target genes were predicted by psRobot with penalty scores ≤2.5 and without mismatched base pairs in the “5′-side” regions (the 2nd to 7th nucleotides of mature miRNA, named as “5′-side” region in this study), and targets were predicted by the TAPIR RNAhybrid engine with scores ≤4 and MFE (minimum free energy) ratios ≥0.7 and without mismatched base pairs in the “5′-side” regions. Merged results from the above two programs were used in the analysis.

ceRNAs were predicted by using the TAPIR RNAhybrid engine with the target decoy search method. Candidates were considered as predicted ceRNAs if they could meet the following criteria: (1) MFE ratio ≥ 0.7, (2) no more than one mismatched base pair between the miRNA “5′-side” region and its recognized region, and (3) a bulge structure in the candidate (a mismatch loop usually 3 nt in length) behind the 10th complementary base pair in the 5′ end of the mature miRNA sequences between the candidate ceRNA and its miRNA.

The parameters for identifying target genes are reasonable based on a previous comparative study of several prediction programs [41], and the rules on structural features used for ceRNA prediction are commonly used in ceRNA identification [23]. Transcriptional isoforms from the same transcribed loci predicted as ceRNA or target genes were merged together to remove redundancy.

### 4.6. Reconstruction of ceRNA-miRNA-Target Gene Regulatory Networks

By using the annotated types of transcripts, the identified known and potentially novel miRNAs, the predicted ceRNA and target relationships, and the expression correlations within ceRNA-miRNAs and miRNA-target pairs, we reconstructed the ceRNA-miRNA-target gene regulatory networks in this study. The miRNA-mediated networks only containing target genes or ceRNAs were filtered out, since no ceRNA and target gene both directly linked to the miRNAs in this analysis. The negative correlations of expression levels (*r* < –0.3) between miRNAs and their target genes, as well as between ceRNAs and their regulated miRNAs at the three anther developmental stages in at least one maize line, were considered as expression evidences and were marked with broader lines in the networks plotted by Cytoscape [92].

### 4.7. Specificity Analysis of Maize Anther Transcriptomes

Maize transcriptome data sets published previously included 79 tissues and developmental stages, and were used to compare differences of transcriptomes between anther and other tissues in maize [36]. Genes specifically expressed in the anther were determined with tissue specificity indexes (TSIs) > 0.9. TSI was calculated for each gene according to the method described previously [93]. A larger TSI value of a certain gene indicates that the gene is more specifically expressed in one of the investigated tissues.

## Supplementary Materials

Supplementary materials can be found at https://www.mdpi.com/1422-0067/20/14/3480/s1.

## Abbreviations

ceRNA	Competing Endogenous RNA
circRNA	circular RNA
CPM	Counts Per Million Mapped Read
GMS	Genic Male Sterility
GRN	Gene Regulatory Network
LncRNA	Long Noncoding RNA
MFE	Minimum Free Energy
miRNA	microRNA
MRE	miRNA Response Element
RNA-seq	RNA-sequencing
SEM	Scanning Electron Microscope
SSR	Simple Sequence Repeat
TE	Transposable Element
TF	Transcription Factor
TSI	Tissue Specificity Index

## References

[1] Goldberg R.B., Beals T.P., Sanders P.M. (1993). Anther development: Basic principles and practical applications. Plant Cell.

[2] Alves-Ferreira M., Wellmer F., Banhara A., Kumar V., Riechmann J.L., Meyerowitz E.M. (2007). Global expression profiling applied to the analysis of *Arabidopsis* stamen development. Plant Physiol..

[3] Reimegard J., Kundu S., Pendle A., Irish V.F., Shaw P., Nakayama N., Sundstrom J.F., Emanuelsson O. (2017). Genome-wide identification of physically clustered genes suggests chromatin-level co-regulation in male reproductive development in *Arabidopsis thaliana*. Nucleic Acids Res..

[4] Wan X., Wu S., Li Z., Dong Z., An X., Ma B., Tian Y., Li J. (2019). Maize genic male-sterility genes and their applications in hybrid breeding: Progress and perspectives. Mol. Plant.

[5] Gomez J.F., Talle B., Wilson Z.A. (2015). Anther and pollen development: A conserved developmental pathway. J. Integr. Plant Biol..

[6] Ge X., Chang F., Ma H. (2010). Signaling and transcriptional control of reproductive development in *Arabidopsis*. Curr. Biol..

[7] Ariizumi T., Toriyama K. (2011). Genetic regulation of sporopollenin synthesis and pollen exine development. Annu. Rev. Plant Biol..

[8] Teotia S., Tang G. (2015). To bloom or not to bloom: Role of microRNAs in plant flowering. Mol. Plant.

[9] Chuck G., Meeley R., Irish E., Sakai H., Hake S. (2007). The maize *tasselseed4* microRNA controls sex determination and meristem cell fate by targeting *Tasselseed6*/*indeterminate spikelet1*. Nat. Genet..

[10] Luo Y., Guo Z., Li L. (2013). Evolutionary conservation of microRNA regulatory programs in plant flower development. Dev. Biol..

[11] Salmena L., Poliseno L., Tay Y., Kats L., Pandolfi P.P. (2011). A ceRNA hypothesis: The rosetta stone of a hidden RNA language?. Cell.

[12] Ebert M.S., Neilson J.R., Sharp P.A. (2007). microRNA sponges: Competitive inhibitors of small RNAs in mammalian cells. Nat. Methods.

[13] Franco-Zorrilla J.M., Valli A., Todesco M., Mateos I., Puga M.I., Rubio-Somoza I., Leyva A., Weigel D., Garcia J.A., Paz-Ares J. (2007). Target mimicry provides a new mechanism for regulation of microRNA activity. Nat. Genet..

[14] Cesana M., Cacchiarelli D., Legnini I., Santini T., Sthandier O., Chinappi M., Tramontano A., Bozzoni I. (2011). A long noncoding RNA controls muscle differentiation by functioning as a competing endogenous RNA. Cell.

[15] Zhu M., Liu J., Xiao J., Yang L., Cai M., Shen H., Chen X., Ma Y., Hu S., Wang Z. (2017). Lnc-mg is a long non-coding RNA that promotes myogenesis. Nat. Commun..

[16] Song Y.X., Sun J.X., Zhao J.H., Yang Y.C., Shi J.X., Wu Z.H., Chen X.W., Gao P., Miao Z.F., Wang Z.N. (2017). Non-coding RNAs participate in the regulatory network of CLDN4 via ceRNA mediated miRNA evasion. Nat. Commun..

[17] Poliseno L., Salmena L., Zhang J., Carver B., Haveman W.J., Pandolfi P.P. (2010). A coding-independent function of gene and pseudogene mRNAs regulates tumour biology. Nature.

[18] Zheng Q., Bao C., Guo W., Li S., Chen J., Chen B., Luo Y., Lyu D., Li Y., Shi G. (2016). Circular RNA profiling reveals an abundant circHIPK3 that regulates cell growth by sponging multiple miRNAs. Nat. Commun..

[19] Cho J., Paszkowski J. (2017). Regulation of rice root development by a retrotransposon acting as a microRNA sponge. eLife.

[20] Witkos T.M., Krzyzosiak W.J., Fiszer A., Koscianska E. (2018). A potential role of extended simple sequence repeats in competing endogenous RNA crosstalk. RNA Biol..

[21] Tay Y., Kats L., Salmena L., Weiss D., Tan S.M., Ala U., Karreth F., Poliseno L., Provero P., Di Cunto F. (2011). Coding-independent regulation of the tumor suppressor *PTEN* by competing endogenous mRNAs. Cell.

[22] Glenfield C., McLysaght A. (2018). Pseudogenes provide evolutionary evidence for the competitive endogenous RNA hypothesis. Mol. Biol. Evol..

[23] Paschoal A.R., Lozada-Chavez I., Domingues D.S., Stadler P.F. (2018). ceRNAs in plants: Computational approaches and associated challenges for target mimic research. Brief. Bioinform..

[24] Thomson D.W., Dinger M.E. (2016). Endogenous microRNA sponges: Evidence and controversy. Nat. Rev. Genet..

[25] Tay Y., Rinn J., Pandolfi P.P. (2014). The multilayered complexity of ceRNA crosstalk and competition. Nature.

[26] Zhao Y., Tang L., Li Z., Jin J., Luo J., Gao G. (2015). Identification and analysis of unitary loss of long-established protein-coding genes in Poaceae shows evidences for biased gene loss and putatively functional transcription of relics. BMC Evol. Biol..

[27] Khemka N., Singh V.K., Garg R., Jain M. (2016). Genome-wide analysis of long intergenic non-coding RNAs in chickpea and their potential role in flower development. Sci. Rep..

[28] Fan C., Hao Z., Yan J., Li G. (2015). Genome-wide identification and functional analysis of lincRNAs acting as miRNA targets or decoys in maize. BMC Genom..

[29] Wang P., Li X., Gao Y., Guo Q., Wang Y., Fang Y., Ma X., Zhi H., Zhou D., Shen W. (2019). LncACTdb 2.0: An updated database of experimentally supported ceRNA interactions curated from low- and high-throughput experiments. Nucleic Acids Res..

[30] Bhattacharya A., Cui Y. (2016). SomamiR 2.0: A database of cancer somatic mutations altering microRNA-ceRNA interactions. Nucleic Acids Res..

[31] Yuan C., Meng X., Li X., Illing N., Ingle R.A., Wang J., Chen M. (2017). PceRBase: A database of plant competing endogenous RNA. Nucleic Acids Res..

[32] Yang X., Zhao Y., Xie D., Sun Y., Zhu X., Esmaeili N., Yang Z., Wang Y., Yin G., Lv S. (2016). Identification and functional analysis of microRNAs involved in the anther development in cotton genic male sterile line Yu98-8A. Int. J. Mol. Sci..

[33] Sun L., Sun G., Shi C., Sun D. (2018). Transcriptome analysis reveals new microRNAs-mediated pathway involved in anther development in male sterile wheat. BMC Genom..

[34] Bai J.F., Wang Y.K., Wang P., Duan W.J., Yuan S.H., Sun H., Yuan G.L., Ma J.X., Wang N., Zhang F.T. (2017). Uncovering male fertility transition responsive miRNA in a wheat photo-thermosensitive genic male sterile line by deep sequencing and degradome analysis. Front. Plant Sci..

[35] Chen J., Su P., Chen P., Li Q., Yuan X., Liu Z. (2018). Insights into the cotton anther development through association analysis of transcriptomic and small RNA sequencing. BMC Plant Biol..

[36] Stelpflug S.C., Sekhon R.S., Vaillancourt B., Hirsch C.N., Buell C.R., De Leon N., Kaeppler S.M. (2016). An expanded maize gene expression atlas based on RNA sequencing and its use to explore root development. Plant Genome.

[37] Nan G.L., Zhai J., Arikit S., Morrow D., Fernandes J., Mai L., Nguyen N., Meyers B.C., Walbot V. (2017). *Ms23*, a master basic helix-loop-helix factor, regulates the specification and development of the tapetum in maize. Development.

[38] Zhai J., Zhang H., Arikit S., Huang K., Nan G.L., Walbot V., Meyers B.C. (2015). Spatiotemporally dynamic, cell-type-dependent premeiotic and meiotic phasiRNAs in maize anthers. Proc. Natl. Acad. Sci. USA.

[39] Wu H.J., Ma Y.K., Chen T., Wang M., Wang X.J. (2012). PsRobot: A web-based plant small RNA meta-analysis toolbox. Nucleic Acids Res..

[40] Bonnet E., He Y., Billiau K., Van de Peer Y. (2010). TAPIR, a web server for the prediction of plant microRNA targets, including target mimics. Bioinformatics.

[41] Srivastava P.K., Moturu T.R., Pandey P., Baldwin I.T., Pandey S.P. (2014). A comparison of performance of plant miRNA target prediction tools and the characterization of features for genome-wide target prediction. BMC Genom..

[42] Chavez Montes R.A., De Fatima Rosas-Cardenas F., De Paoli E., Accerbi M., Rymarquis L.A., Mahalingam G., Marsch-Martinez N., Meyers B.C., Green P.J., De Folter S. (2014). Sample sequencing of vascular plants demonstrates widespread conservation and divergence of microRNAs. Nat. Commun..

[43] You C., Cui J., Wang H., Qi X., Kuo L.Y., Ma H., Gao L., Mo B., Chen X. (2017). Conservation and divergence of small RNA pathways and microRNAs in land plants. Genome Biol..

[44] Yamasaki T., Voshall A., Kim E.J., Moriyama E., Cerutti H., Ohama T. (2013). Complementarity to an miRNA seed region is sufficient to induce moderate repression of a target transcript in the unicellular green alga *Chlamydomonas reinhardtii*. Plant J..

[45] Liu Q., Wang F., Axtell M.J. (2014). Analysis of complementarity requirements for plant microRNA targeting using a *Nicotiana benthamiana* quantitative transient assay. Plant Cell.

[46] Axtell M.J. (2008). Evolution of microRNAs and their targets: Are all microRNAs biologically relevant?. Biochim. Biophys. Acta.

[47] Zhang D., Wu S., An X., Xie K., Dong Z., Zhou Y., Xu L., Fang W., Liu S., Liu S. (2018). Construction of a multicontrol sterility system for a maize male-sterile line and hybrid seed production based on the *Zm**Ms7* gene encoding a PHD-finger transcription factor. Plant Biotechnol. J..

[48] Wang J.W., Czech B., Weigel D. (2009). miR156-regulated SPL transcription factors define an endogenous flowering pathway in *Arabidopsis thaliana*. Cell.

[49] Xing S., Salinas M., Hohmann S., Berndtgen R., Huijser P. (2010). miR156-targeted and nontargeted SBP-box transcription factors act in concert to secure male fertility in *Arabidopsis*. Plant Cell.

[50] Zhu Q.H., Upadhyaya N.M., Gubler F., Helliwell C.A. (2009). Over-expression of miR172 causes loss of spikelet determinacy and floral organ abnormalities in rice (*Oryza sativa*). BMC Plant Biol..

[51] Chuck G., Meeley R., Hake S. (2008). Floral meristem initiation and meristem cell fate are regulated by the maize *AP2* genes *ids1* and *sid1*. Development.

[52] Wu G., Park M.Y., Conway S.R., Wang J.W., Weigel D., Poethig R.S. (2009). The sequential action of miR156 and miR172 regulates developmental timing in *Arabidopsis*. Cell.

[53] Wang H., Mao Y., Yang J., He Y. (2015). *TCP42* modulates secondary cell wall thickening and anther endothecium development. Front. Plant Sci..

[54] Nag A., King S., Jack T. (2009). miR319a targeting of *TCP4* is critical for petal growth and development in *Arabidopsis*. Proc. Natl. Acad. Sci. USA.

[55] Kaneko M., Inukai Y., Ueguchi-Tanaka M., Itoh H., Izawa T., Kobayashi Y., Hattori T., Miyao A., Hirochika H., Ashikari M. (2004). Loss-of-function mutations of the rice *GAMYB* gene impair α-amylase expression in aleurone and flower development. Plant Cell.

[56] Yang J., Tian L., Sun M.X., Huang X.Y., Zhu J., Guan Y.F., Jia Q.S., Yang Z.N. (2013). *AUXIN RESPONSE FACTOR17* is essential for pollen wall pattern formation in *Arabidopsis*. Plant Physiol..

[57] Yu J., Meng Z., Liang W., Behera S., Kudla J., Tucker M.R., Luo Z., Chen M., Xu D., Zhao G. (2016). A rice Ca^2+^ binding protein is required for tapetum function and pollen formation. Plant Physiol..

[58] Zhao G., Shi J., Liang W., Xue F., Luo Q., Zhu L., Qu G., Chen M., Schreiber L., Zhang D. (2015). Two ATP binding cassette G transporters, rice ATP binding cassette G26 and ATP binding cassette G15, collaboratively regulate rice male reproduction. Plant Physiol..

[59] Ito T., Shinozaki K. (2002). The *MALE STERILITY1* gene of *Arabidopsis*, encoding a nuclear protein with a PHD-finger motif, is expressed in tapetal cells and is required for pollen maturation. Plant Cell Physiol..

[60] Li H., Yuan Z., Vizcay-Barrena G., Yang C., Liang W., Zong J., Wilson Z.A., Zhang D. (2011). Persistent tapetal cell1 encodes a PHD-finger protein that is required for tapetal cell death and pollen development in rice. Plant Physiol..

[61] Li N., Zhang D.S., Liu H.S., Yin C.S., Li X.X., Liang W.Q., Yuan Z., Xu B., Chu H.W., Wang J. (2006). The rice tapetum degeneration retardation gene is required for tapetum degradation and anther development. Plant Cell.

[62] Zhang D.S., Liang W.Q., Yuan Z., Li N., Shi J., Wang J., Liu Y.M., Yu W.J., Zhang D.B. (2008). Tapetum degeneration retardation is critical for aliphatic metabolism and gene regulation during rice pollen development. Mol. Plant.

[63] Schwanhausser B., Busse D., Li N., Dittmar G., Schuchhardt J., Wolf J., Chen W., Selbach M. (2011). Global quantification of mammalian gene expression control. Nature.

[64] Wan X., Wu S. (2019). Molecular cloning of genic male-sterility genes and their applications for plant heterosis via biotechnology-based male-sterility systems. Molecular Cloning—Methods and Applications.

[65] Wan X., Li Z. (2019). Plant comparative transcriptomics reveals functional mechanisms and gene regulatory networks involved in anther development and male sterility. Plant Genomics and Transcriptomics.

[66] Li Z.W., Chen X., Wu Q., Hagmann J., Han T.S., Zou Y.P., Ge S., Guo Y.L. (2016). On the origin of *de novo* genes in *Arabidopsis thaliana* populations. Genome Biol. Evol..

[67] Li Z., Wan X. (2018). Long-term evolutionary DNA methylation dynamic of protein-coding genes and its underlying mechanism. Gene.

[68] Xie K., Wu S., Li Z., Zhou Y., Zhang D., Dong Z., An X., Zhu T., Zhang S., Liu S. (2018). Map-based cloning and characterization of *Zea mays male sterility33* (*Zm**Ms33*) gene, encoding a glycerol-3-phosphate acyltransferase. Theor. Appl. Genet..

[69] An X., Dong Z., Tian Y., Xie K., Wu S., Zhu T., Zhang D., Zhou Y., Niu C., Ma B. (2019). *Zm**Ms30* encoding a novel GDSL lipase is essential for male fertility and valuable for hybrid breeding in maize. Mol. Plant.

[70] Zhu T., Wu S., Zhang D., Li Z., Xie K., An X., Ma B., Hou Q., Dong Z., Tian Y. (2019). Genome-wide analysis of maize *GPAT* gene family and cytological characterization and breeding application of *Zm**Ms33*/*Zm**GPAT6* gene. Theor. Appl. Genet..

[71] Wang Y., Liu D., Tian Y., Wu S., An X., Dong Z., Zhang S., Bao J., Li Z., Li J. (2019). Map-based cloning, phylogenetic, and microsynteny analyses of *Zm**Ms20* gene regulating male fertility in maize. Int. J. Mol. Sci..

[72] Nan G.L., Ronceret A., Wang R.C., Fernandes J.F., Cande W.Z., Walbot V. (2011). Global transcriptome analysis of two *ameiotic1* alleles in maize anthers: Defining steps in meiotic entry and progression through prophase I. BMC Plant Biol..

[73] Patel R.K., Jain M. (2012). NGS QC Toolkit: A toolkit for quality control of next generation sequencing data. PLoS ONE.

[74] Trapnell C., Pachter L., Salzberg S.L. (2009). TopHat: Discovering splice junctions with RNA-seq. Bioinformatics.

[75] Trapnell C., Williams B.A., Pertea G., Mortazavi A., Kwan G., Van Baren M.J., Salzberg S.L., Wold B.J., Pachter L. (2010). Transcript assembly and quantification by RNA-seq reveals unannotated transcripts and isoform switching during cell differentiation. Nature Biotechnol..

[76] Robinson M.D., McCarthy D.J., Smyth G.K. (2010). edgeR: A Bioconductor package for differential expression analysis of digital gene expression data. Bioinformatics.

[77] McKenna A., Hanna M., Banks E., Sivachenko A., Cibulskis K., Kernytsky A., Garimella K., Altshuler D., Gabriel S., Daly M. (2010). The Genome Analysis Toolkit: A MapReduce framework for analyzing next-generation DNA sequencing data. Genome Res..

[78] Haas B.J., Papanicolaou A., Yassour M., Grabherr M., Blood P.D., Bowden J., Couger M.B., Eccles D., Li B., Lieber M. (2013). *De novo* transcript sequence reconstruction from RNA-seq using the Trinity platform for reference generation and analysis. Nat. Protoc..

[79] Grabherr M.G., Haas B.J., Yassour M., Levin J.Z., Thompson D.A., Amit I., Adiconis X., Fan L., Raychowdhury R., Zeng Q. (2011). Full-length transcriptome assembly from RNA-seq data without a reference genome. Nat. Biotechnol..

[80] Altschul S.F., Madden T.L., Schaffer A.A., Zhang J.H., Zhang Z., Miller W., Lipman D.J. (1997). Gapped BLAST and PSI-BLAST: A new generation of protein database search programs. Nucleic Acids Res..

[81] Boerner S., McGinnis K.M. (2012). Computational identification and functional predictions of long noncoding RNA in *Zea mays*. PLoS ONE.

[82] Li L., Eichten S.R., Shimizu R., Petsch K., Yeh C.T., Wu W., Chettoor A.M., Givan S.A., Cole R.A., Fowler J.E. (2014). Genome-wide discovery and characterization of maize long non-coding RNAs. Genome Biol..

[83] Wang H., Niu Q.W., Wu H.W., Liu J., Ye J., Yu N., Chua N.H. (2015). Analysis of non-coding transcriptome in rice and maize uncovers roles of conserved lncRNAs associated with agriculture traits. Plant J..

[84] Zhang W., Han Z., Guo Q., Liu Y., Zheng Y., Wu F., Jin W. (2014). Identification of maize long non-coding RNAs responsive to drought stress. PLoS ONE.

[85] Zhang Z., Carriero N., Zheng D., Karro J., Harrison P.M., Gerstein M. (2006). PseudoPipe: An automated pseudogene identification pipeline. Bioinformatics.

[86] Nawrocki E.P., Eddy S.R. (2013). Infernal 1.1: 100-fold faster RNA homology searches. Bioinformatics.

[87] Kalvari I., Argasinska J., Quinones-Olvera N., Nawrocki E.P., Rivas E., Eddy S.R., Bateman A., Finn R.D., Petrov A.I. (2018). Rfam 13.0: Shifting to a genome-centric resource for non-coding RNA families. Nucleic Acids Res..

[88] Kalvari I., Nawrocki E.P., Argasinska J., Quinones-Olvera N., Finn R.D., Bateman A., Petrov A.I. (2018). Non-coding RNA analysis using the Rfam database. Curr. Protoc. Bioinform..

[89] Langmead B., Trapnell C., Pop M., Salzberg S.L. (2009). Ultrafast and memory-efficient alignment of short DNA sequences to the human genome. Genome Biol..

[90] Kozomara A., Griffiths-Jones S. (2014). miRBase: Annotating high confidence microRNAs using deep sequencing data. Nucleic Acids Res..

[91] Friedlander M.R., Mackowiak S.D., Li N., Chen W., Rajewsky N. (2012). miRDeep2 accurately identifies known and hundreds of novel microRNA genes in seven animal clades. Nucleic Acids Res..

[92] Shannon P., Markiel A., Ozier O., Baliga N.S., Wang J.T., Ramage D., Amin N., Schwikowski B., Ideker T. (2003). Cytoscape: A software environment for integrated models of biomolecular interaction networks. Genome Res..

[93] Yanai I., Benjamin H., Shmoish M., Chalifa-Caspi V., Shklar M., Ophir R., Bar-Even A., Horn-Saban S., Safran M., Domany E. (2005). Genome-wide midrange transcription profiles reveal expression level relationships in human tissue specification. Bioinformatics.

