# Supplementary material for: Discovering and Constructing ceRNA-miRNA-Target Gene Regulatory Networks during Anther Development in Maize
Source: Int J Mol Sci. 2019 Jul 15;20(14):3480. doi: 10.3390/ijms20143480 (PMC6678786; doi:10.3390/ijms20143480)
Supplement: Supplementary file 1 [file ijms-20-03480-s001.zip › ijms-543828-proof done-supplementary/Supporting Figure S1.pdf]

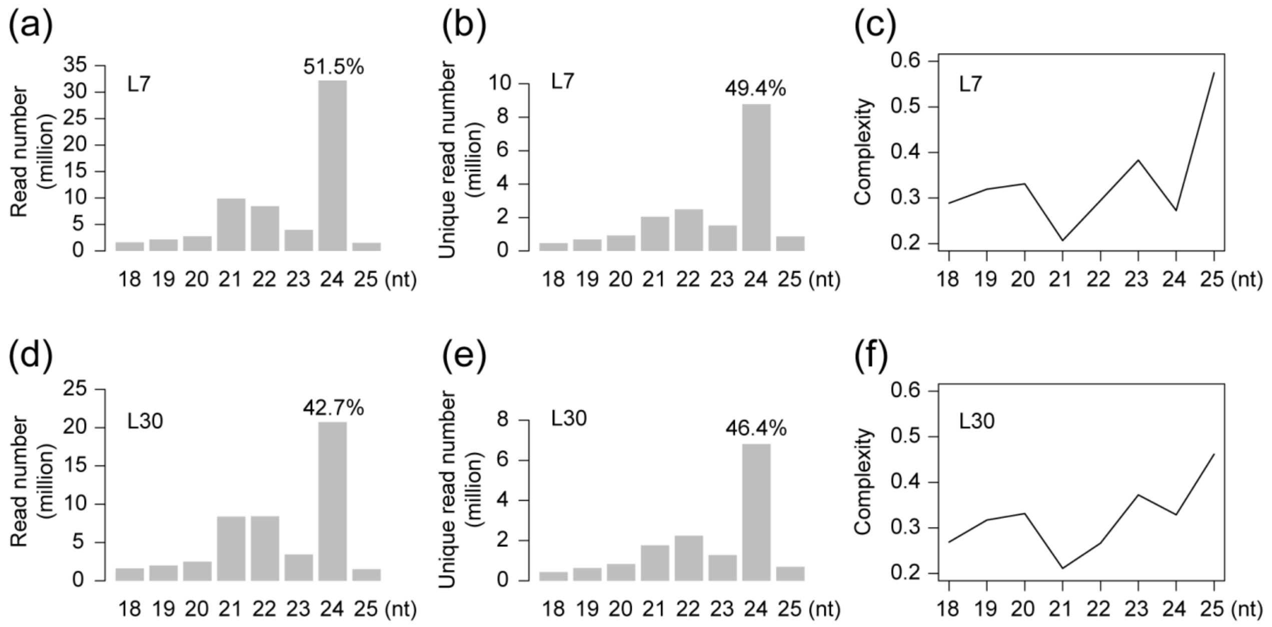

Figure S1. Length distribution of reads in the small RNA-seq data of maize lines L7 and L30.

We estimated both the numbers of all reads (**a** and **d**) and the unique reads (**b** and **e**) ranging from 18 to 25 nt in length. “Complexity” (**c** and **f**) was the ratio between the number of unique reads and the number of all reads for each length class.
