# Supplementary material for: Discovering and Constructing ceRNA-miRNA-Target Gene Regulatory Networks during Anther Development in Maize
Source: Int J Mol Sci. 2019 Jul 15;20(14):3480. doi: 10.3390/ijms20143480 (PMC6678786; doi:10.3390/ijms20143480)
Supplement: Supplementary file 1 [file ijms-20-03480-s001.zip › ijms-543828-proof done-supplementary/Supporting Figure S2.pdf]

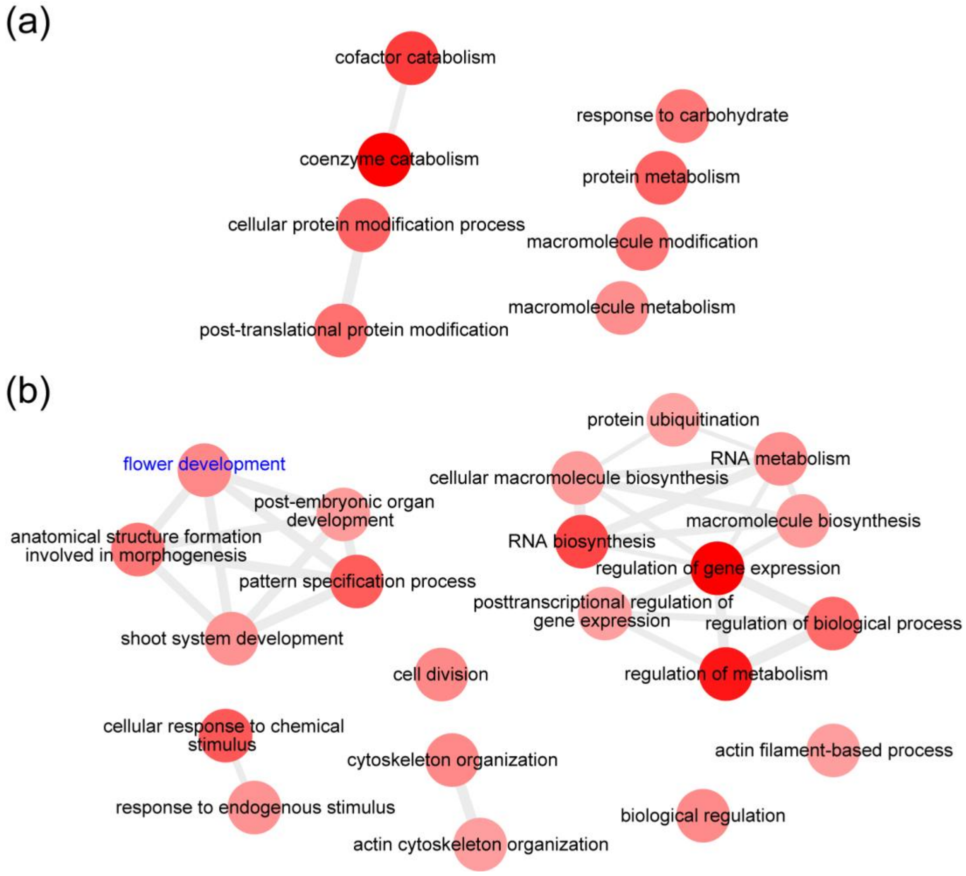

Figure S2. GO enrichment analysis.

Results of **a** ceRNAs and **b** target genes in "biological process" category were shown. GO term of "flower development" was shown in blue. REViGO (http://revigo.irb.hr/) was used to remove redundancy in GO terms.
