# Supplementary material for: Discovering and Constructing ceRNA-miRNA-Target Gene Regulatory Networks during Anther Development in Maize
Source: Int J Mol Sci. 2019 Jul 15;20(14):3480. doi: 10.3390/ijms20143480 (PMC6678786; doi:10.3390/ijms20143480)
Supplement: Supplementary file 1 [file ijms-20-03480-s001.zip › ijms-543828-proof done-supplementary/Supporting Figure S3.pdf]

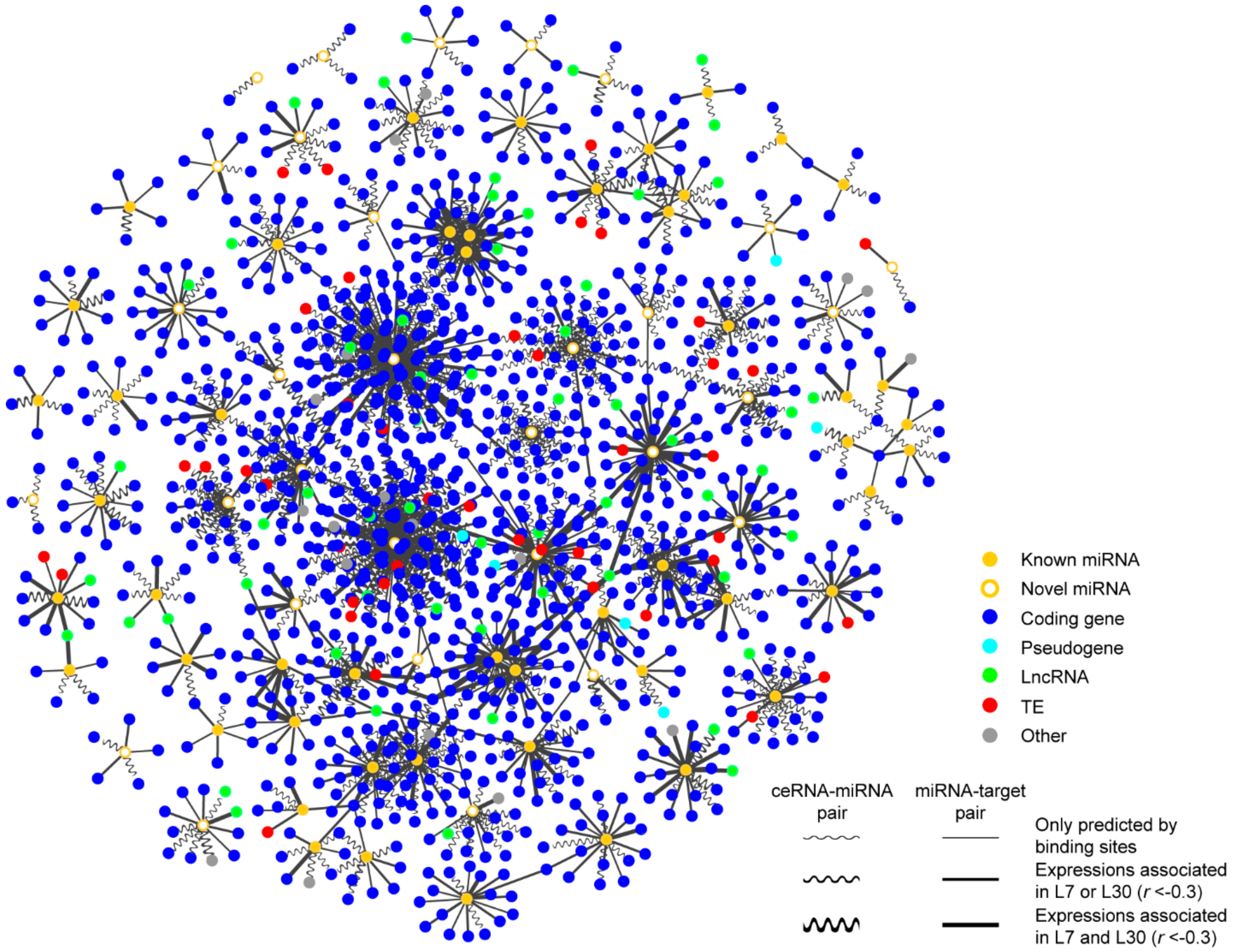

Figure S3. A diagram of reconstructed ceRNA-miRNA-target gene regulatory networks and their crosstalk.

Known and potentially novel miRNAs identified in this study, and the predicted ceRNAs and target genes, were all included in the network. Different types of transcribed loci were marked in various colors. ceRNA-miRNA pairs were shown linked by wavy lines, while miRNA-target pairs were linked by straight lines. The number of maize lines (none, one, or two) with negative expression correlations in each ceRNA-miRNA pair or miRNA-target pair was represented by the line width.
