# Supplementary material for: Discovering and Constructing ceRNA-miRNA-Target Gene Regulatory Networks during Anther Development in Maize
Source: Int J Mol Sci. 2019 Jul 15;20(14):3480. doi: 10.3390/ijms20143480 (PMC6678786; doi:10.3390/ijms20143480)
Supplement: Supplementary file 1 [file ijms-20-03480-s001.zip › ijms-543828-proof done-supplementary/Supporting Figure S4.pdf]

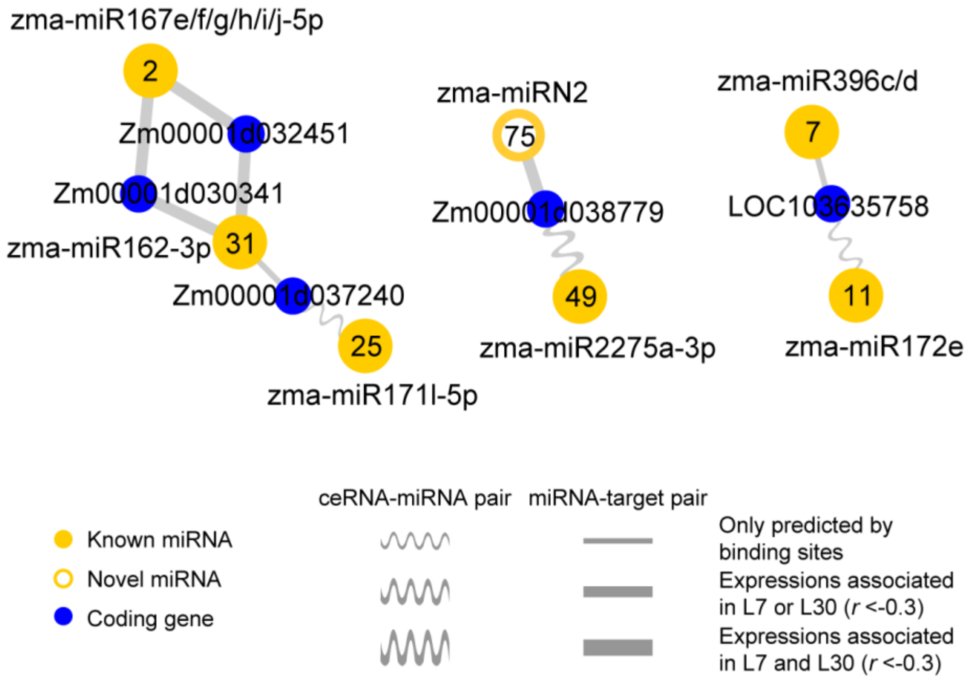

Figure S4. The relatively simple crosstalk between reconstructed ceRNA-miRNA-target gene regulatory networks.

Numbers in the yellow dots and circles represent known and potential novel miRNAs numbered in Figure 4.
