# Supplementary material for: Discovering and Constructing ceRNA-miRNA-Target Gene Regulatory Networks during Anther Development in Maize
Source: Int J Mol Sci. 2019 Jul 15;20(14):3480. doi: 10.3390/ijms20143480 (PMC6678786; doi:10.3390/ijms20143480)
Supplement: Supplementary file 1 [file ijms-20-03480-s001.zip › ijms-543828-proof done-supplementary/Supporting Figure S5.pdf]

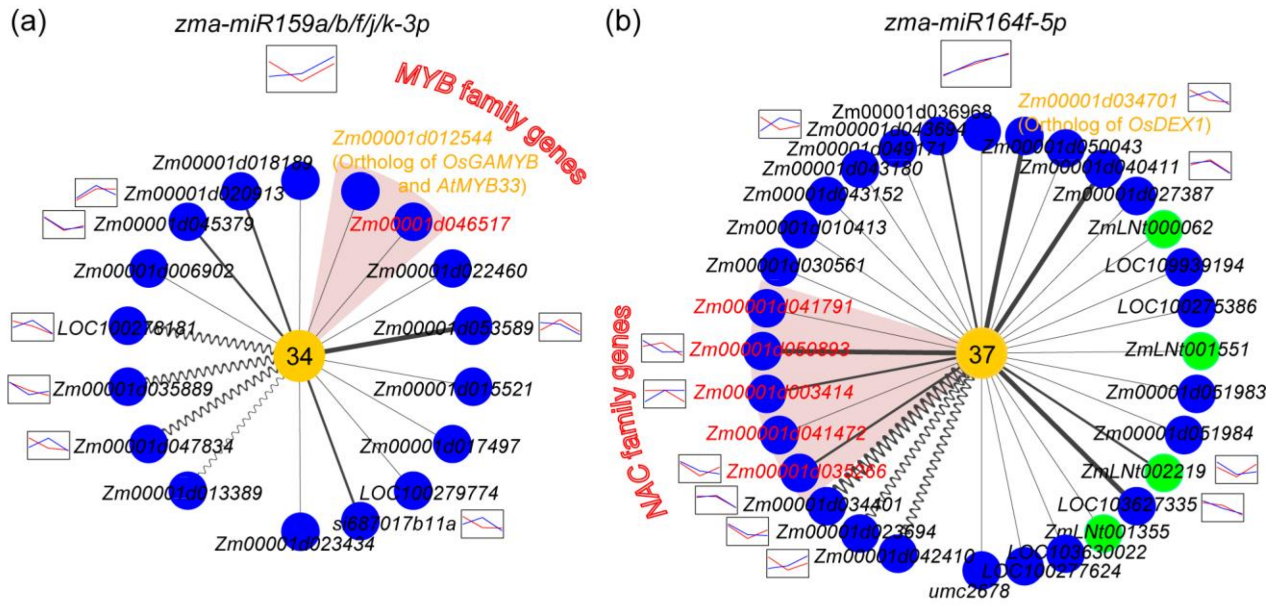

Figure S5. Paralogous miRNA-mediated ceRNA-miRNA-target gene regulatory networks associated with anther development.

Networks of **a** zma-miR159 and **b** zma-miR164 paralogs were shown The transcription patterns of the corresponding miRNAs were shown at the top of the networks based on small RNA transcriptomes in maize lines L7 (red curve) and L30 (blue curve). Transcription patterns of genes negatively associated with miRNA expression in at least one of the two lines were shown beside the gene names. The names and backgrounds of genes in the same gene family in the network were marked in red. The names of genes with orthologs that participate in anther development in other plant species were shown in yellow. Numbers in the yellow dots represent known miRNAs numbered in Figure 4.
