# Supplementary material for: Discovering and Constructing ceRNA-miRNA-Target Gene Regulatory Networks during Anther Development in Maize
Source: Int J Mol Sci. 2019 Jul 15;20(14):3480. doi: 10.3390/ijms20143480 (PMC6678786; doi:10.3390/ijms20143480)
Supplement: Supplementary file 1 [file ijms-20-03480-s001.zip › ijms-543828-proof done-supplementary/Supporting Figure S6.pdf]

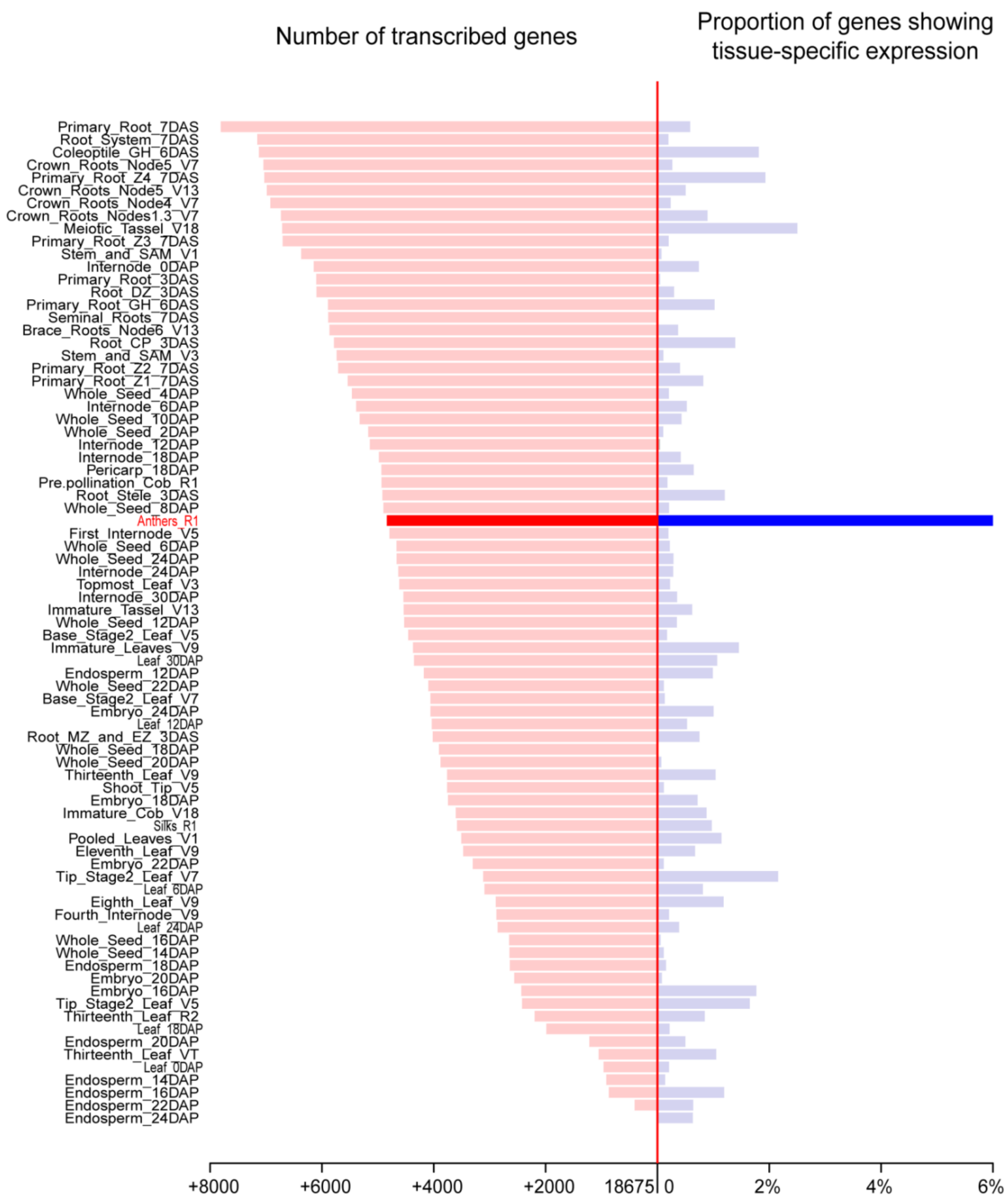

Figure S6. Proportions of transcribed genes that show tissue-specificity in maize transcriptomes.

The left part of the figure shows the numbers of transcribed genes in different maize tissues at various time points, while the right part shows the proportion of tissue-specific transcribed genes in the total number of transcribed genes for each tissue. There were ~23,000 transcribed genes (marked in dark red) in the anther transcriptomes and ~6% of them (marked in dark blue) were specifically transcribed in the anther. This proportion was the highest among the 79 tissues sampled at different developmental stages.
